# Supplementary material for: Downregulated NDR1 protein kinase inhibits innate immune response by initiating an miR146a-STAT1 feedback loop
Source: Nat Commun. 2018 Jul 17;9:2789. doi: 10.1038/s41467-018-05176-7 (PMC6050289; doi:10.1038/s41467-018-05176-7)
Supplement: Supplementary file 1 — Supplementary Information [file 41467_2018_5176_MOESM1_ESM.pdf]

Downregulated NDR1 protein kinase inhibits innate immune response by initiating an miR146a-STAT1 feedback loop

Liu et al.

Supplementary Figures and legends

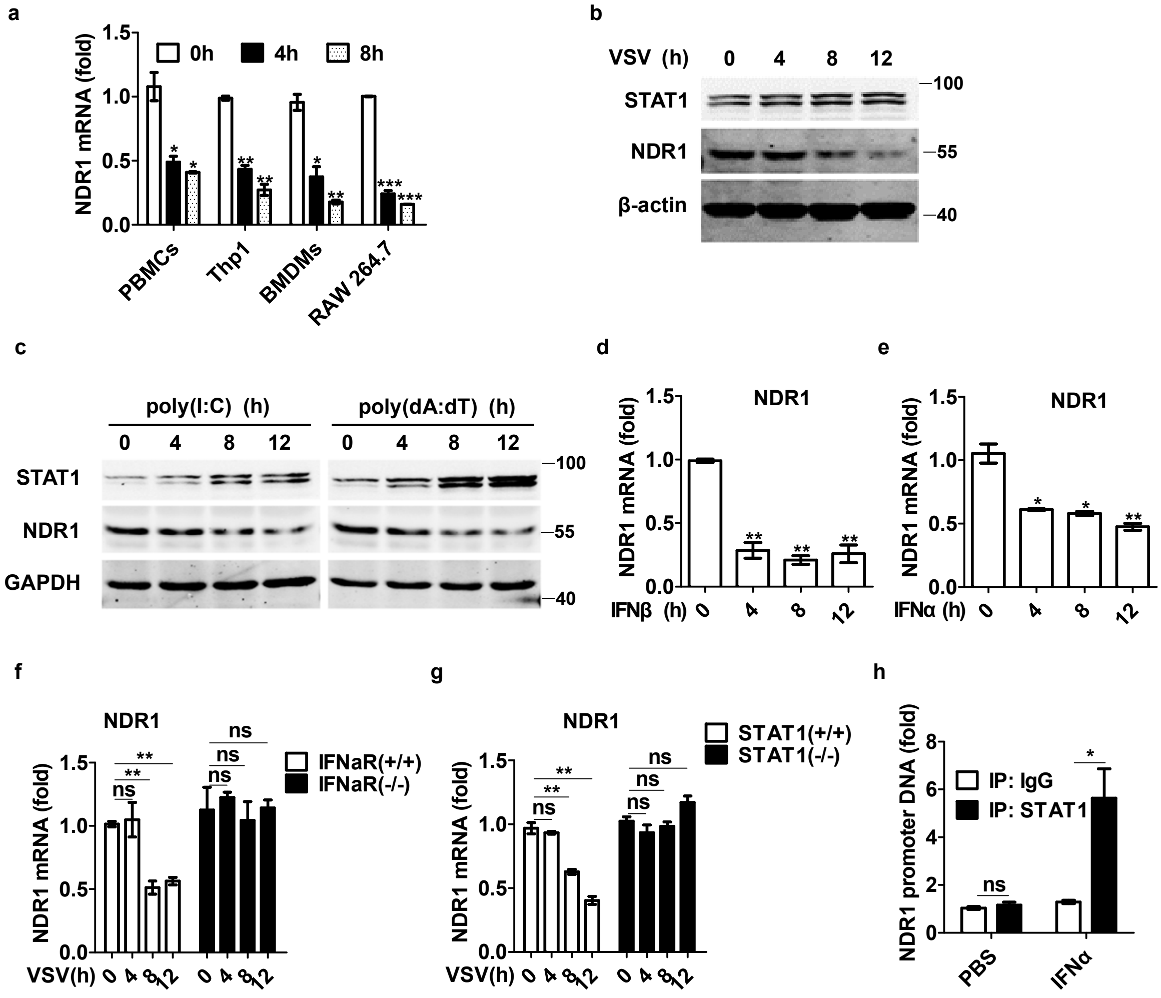

Supplementary Figure 1. Viral infection downregulates NDR1 expression in a type I interferon pathway-dependent manner.

(**a**) Real-time PCR analysis of NDR1 mRNA in human primary peripheral blood monocytes (PBMCs), Thp1 cells, mouse bone marrow derived macrophages (BMDMs) and RAW264.7 cell line infected with VSV.

(**b**) Immunoblot analysis of the dynamic expression of NDR1 in lysates of Thp1 cells infected with VSV for the indicated times.

(**c**) Immunoblot analysis of the dynamic expression of NDR1 in lysates of peritoneal macrophages (PMs) transfected with ploy(I:C) (1 μg ml^{-1}) or poly(dA:dT) (1 μg ml^{-1}).

(**d,e**) Real-time PCR analysis of NDR1 mRNA in PMs treated with IFNβ (100 IU ml^{-1}) or Thp1 cells treated with IFNα (100 IU ml^{-1}).

(**f,g**) Real-time PCR analysis of NDR1 mRNA in *IFNαR*$^{+/+}$ and *IFNαR*$^{-/-}$ (**f**) or *STAT1*$^{+/+}$ *and STAT1*$^{-/-}$ (**g**) mouse immortalized BMDMs infected with VSV.

(**h**) ChIP-qPCR analysis of the enrichment of STAT1 to NDR1 promoter with anti-STAT1 antibody in lysates of Thp1 cells treated with PBS or IFNα (100 IU ml^{-1}) for 4 h. Data are mean ± SD and are representative of three independent experiments. Student's t-test was used for statistical calculation. *$p<0.05$, **$p<0.01$, ***$p < 0.001$.

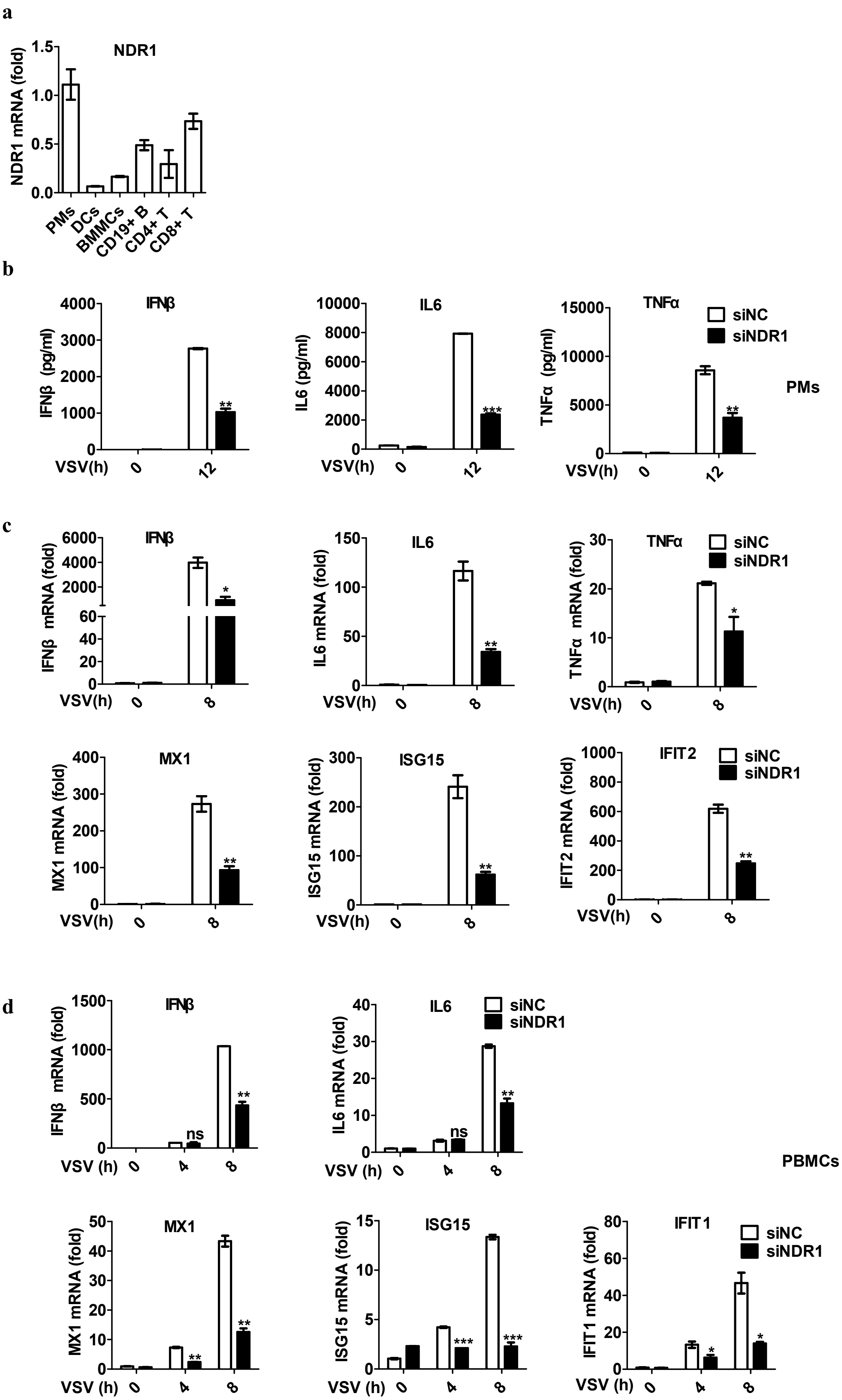
a
NDR1
NDR1 mRNA (fold)
1.5
1.0
0.5
0.0
PMs
DCs
BMMCs
CD19+ B
CD4+ T
CD8+ T
b
IFNβ
IFNβ (pg/ml)
4000
3000
2000
1000
0
VSV(h)
0
12
**
IL6
IL6 (pg/ml)
10000
8000
6000
4000
2000
0
***
TNFα
TNFα (pg/ml)
15000
10000
5000
**
siNC
siNDR1
PMs
c
IFNβ
IFNβ mRNA (fold)
6000
4000
2000
60
40
20
0
8
*
IL6
IL6 mRNA (fold)
150
100
50
**
TNFα
TNFα mRNA (fold)
30
20
10
*
MX1
MX1 mRNA (fold)
400
300
200
100
**
ISG15
ISG15 mRNA (fold)
300
200
100
**
IFIT2
IFIT2 mRNA (fold)
1000
800
600
400
200
**
d
IFNβ
IFNβ mRNA (fold)
1500
1000
500
VSV (h)
4
ns
**
IL6
IL6 mRNA (fold)
40
30
20
10
ns
**
PBMCs
MX1
MX1 mRNA (fold)
50
40
30
20
10
**
ISG15
ISG15 mRNA (fold)
15
10
5
***
IFIT1
IFIT1 mRNA (fold)
80
60
40
20
*

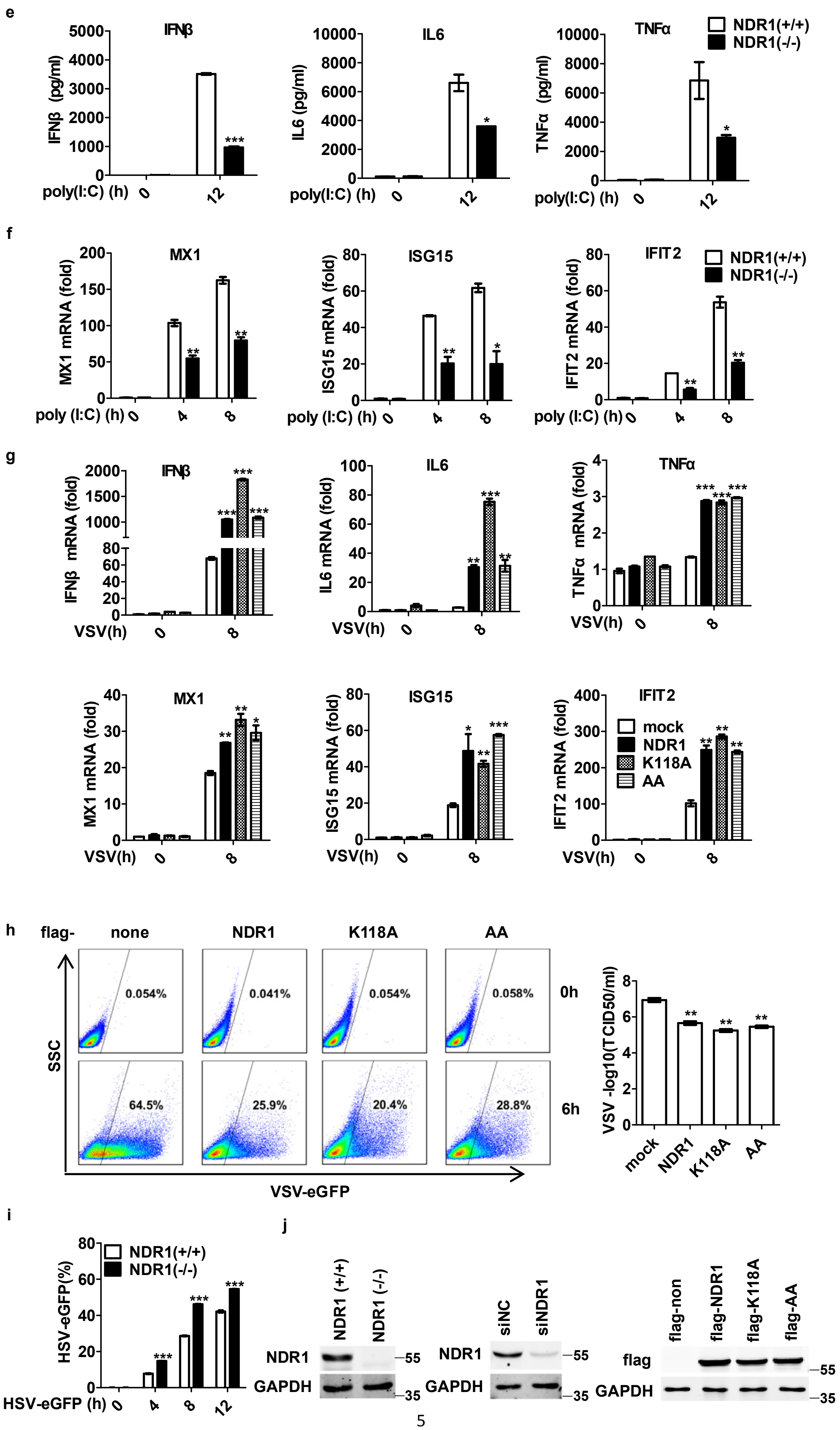
e
IFNβ
IFNβ (pg/ml)
IL6
IL6 (pg/ml)
TNFα
TNFα (pg/ml)
NDR1(+/+)
NDR1(-/-)
poly(I:C) (h)
f
MX1
MX1 mRNA (fold)
ISG15
ISG15 mRNA (fold)
IFIT2
IFIT2 mRNA (fold)
poly (I:C) (h)
g
IFNβ mRNA (fold)
IL6 mRNA (fold)
TNFα mRNA (fold)
VSV(h)
mock
NDR1
K118A
AA
h
flag-
none
NDR1
K118A
AA
0.054%
0.041%
0.054%
0.058%
0h
64.5%
25.9%
20.4%
28.8%
6h
SSC
VSV-eGFP
VSV -log10(TCID50/ml)
i
HSV-eGFP(%)
HSV-eGFP (h)
j
NDR1 (+/+)
NDR1 (-/-)
NDR1
GAPDH
siNC
siNDR1
flag-non
flag-NDR1
flag-K118A
flag-AA
flag
55
35

Supplementary Figure 2. NDR1 promotes viral infection-induced IFNβ, proinflammatory cytokines and antiviral ISGs production in macrophages in a kinase-independent manner.

(**a**) Real-time PCR analysis of NDR1 mRNA in mouse immune cells including primary PMs, dendritic cells, bone marrow-derived mast cells (BMMCs), $CD19^+$ B cells, $CD4^+$ T cells and $CD8^+$ T cells.

(**b**) ELISA of IFNβ, IL6 and TNFα protein in PMs treated with NDR1-specific or scrambled siRNA followed by VSV infection.

(**c**) Real-time PCR analysis of IFNβ, IL6, TNFα and antiviral ISGs mRNA in PMs treated with NDR1-specific or scrambled siRNA for 48h, followed by VSV infection.

(**d**) Real-time PCR analysis of IFNβ, IL6 and antiviral ISGs mRNA in human PBMCs treated with NDR1-specific or scrambled siRNA for 48h, followed by VSV infection.

(**e,f**) ELISA of IFNβ, IL6 and TNFα protein **(e)** and real-time PCR analysis of antiviral ISGs mRNA (**f**) in wild-type and *NDR1*-deficient PMs transfected with poly(I:C) (1 $\mu g\ ml^{-1}$).

(**g**) Real-time PCR analysis of IFNβ, IL6, TNFα and antiviral ISGs mRNA in RAW264.7 cells stably overexpressing NDR1 and its kinase-inactive mutants infected with VSV.

(**h**) FACS analysis of RAW264.7 cells stably overexpressing NDR1 and its kinase-inactive mutants infected with VSV-eGFP. The fresh medium with the VSV-eGFP infection was collected after 12 h for TCID 50 assessing.

(**i**) FACS analysis of wild-type and *NDR1*-deficient PMs infected with HSV-eGFP.

(**j**) Immunoblot analysis of the expression of NDR1 protein in primary PMs with NDR1 knockout or knockdown, or RAW264.7 transfectants stably overexpressing NDR1 or its kinase-inactive mutants. Data are mean ± SD and are representative of three independent experiments. Student's t-test was used for statistical calculation. *$p<0.05$, **$p<0.01$, ***$p < 0.001$.

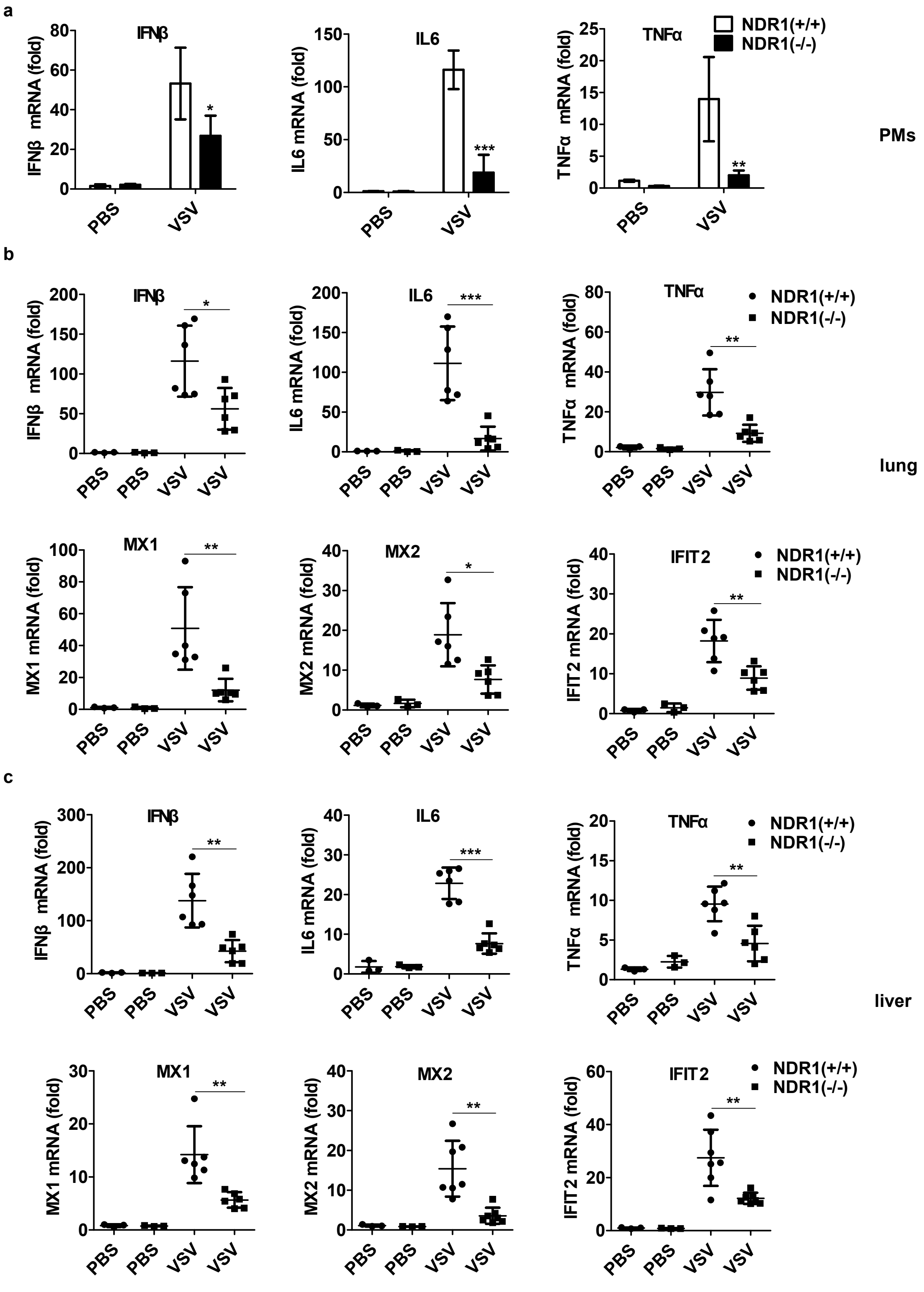
a
b
c
IFNβ
IL6
TNFα
MX1
MX2
IFIT2
NDR1(+/+)
NDR1(-/-)
IFNβ mRNA (fold)
IL6 mRNA (fold)
TNFα mRNA (fold)
MX1 mRNA (fold)
MX2 mRNA (fold)
IFIT2 mRNA (fold)
PBS
VSV
PMs
lung
liver

d

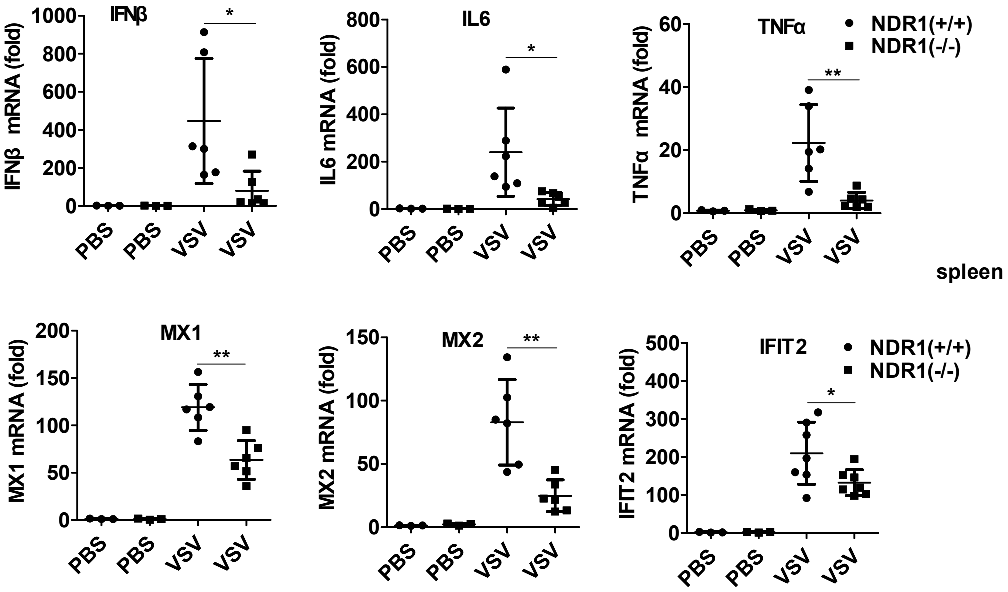

Supplementary Figure 3. NDR1 deficiency inhibits VSV infection-induced IFNβ, proinflammatory cytokines and antiviral ISGs expression *in vivo.*

(**a**) Real-time PCR analysis of IFNβ, IL6 and TNFα mRNA in PMs obtained from 8-weeks-old male $NDR1^{+/+}$ and $NDR1^{-/-}$ mice 12 h after intraperitoneally injected with VSV (1×10^7 pfu g^{-1}) (n = 6 per group). All mice were housed in SPF conditions.

(**b-d**) Real-time PCR analysis of IFNβ, IL6, TNFα and antiviral ISGs mRNA in lung (**b**), liver (**c**) and spleen (**d**) from $NDR1^{+/+}$ and $NDR1^{-/-}$ mice treated as in (**a**). Data are mean ± SD and are representative of three independent experiments. Student's t-test was used for statistical calculation. *p<0.05, **p<0.01, ***p < 0.001.

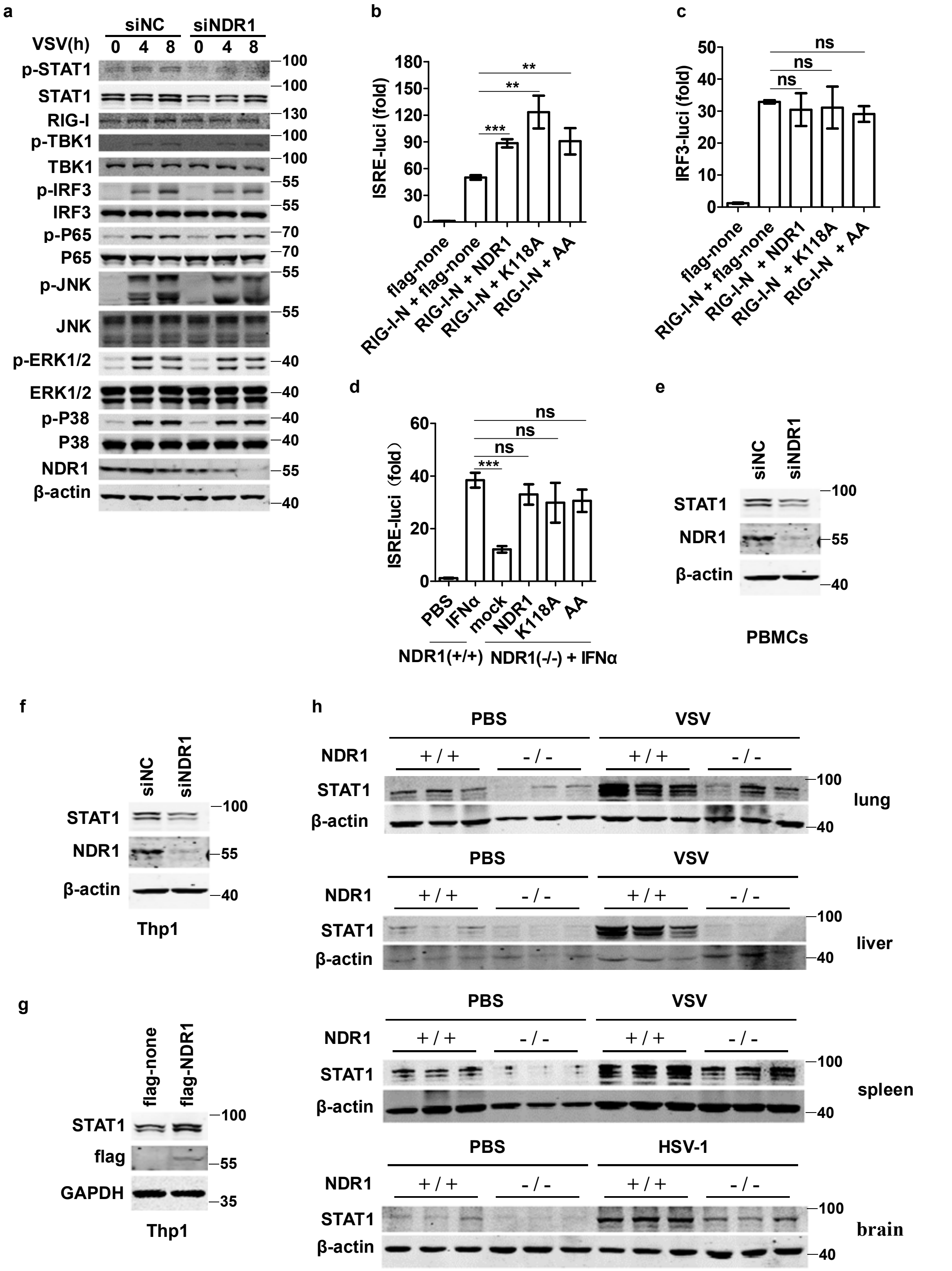

Supplementary Figure 4. NDR1 enhances the expression of STAT1 in a kinase-independent manner.

(**a**) Immunoblot analysis of total and phosphorylated (p-) STAT1, TBK1, IRF3, P65, ERK1/2, JNK1/2 and P38 in lysates of PMs treated with NDR1-specific or scrambled siRNA, followed by VSV

infection.

(**b**,**c**) Luciferase activity in HEK293 cells transfected with plasmids encoding a luciferase reporter for ISRE (**b**) or IRF3 (**c**), and an expressing vector for NDR1 or its kinase-inactive mutants, together with plasmids encoding RIG-I CARD.

(**d**) Luciferase activity in wild-type and *NDR1*$^{-/-}$ HEK293 cells transfected with plasmids encoding a luciferase reporter for ISRE and an expression vector for NDR1 or its kinase-inactive mutants, followed by stimulation with IFNα (100 IU ml^{-1}) for 30 h.

(**e,f**) Immunoblot analysis of STAT1 in primary human PBMCs (**e**) or Thp1 cells (**f**) treated with NDR1-specific or scrambled siRNA for 48 h.

(**g**) Immunoblot analysis of STAT1 expression in Thp1 cells stably overexpressing flag-none or flag-NDR1.

(**h**) Immunoblot analysis of STAT1 expression in lung, liver and spleen obtained from *NDR1*$^{+/+}$ and *NDR1*$^{-/-}$ mice 12 h after intraperitoneally injected with PBS or VSV (1×10^7 pfu g^{-1}) or in brain obtained from *NDR1*$^{+/+}$ and *NDR1*$^{-/-}$ mice intravenously injected with PBS or HSV for 4 days (1×10^6 pfu per mouse). Data are mean ± SD and are representative of three independent experiments. Student's t-test was used for statistical calculation. $^{**}p<0.01$, $^{***}p < 0.001$.

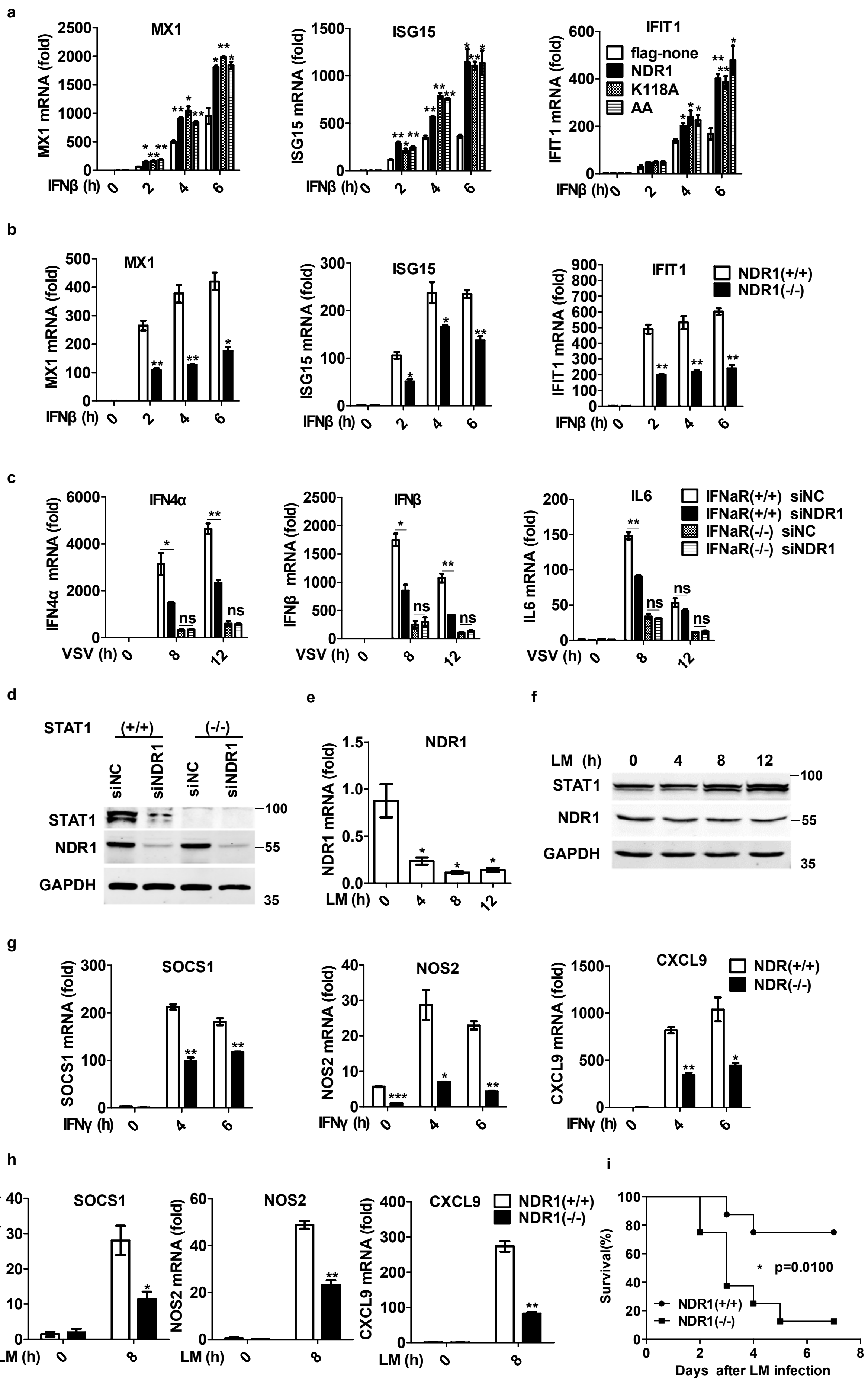
a
MX1
MX1 mRNA (fold)
IFNβ (h)
ISG15
ISG15 mRNA (fold)
IFIT1
IFIT1 mRNA (fold)
flag-none
NDR1
K118A
AA
b
MX1
MX1 mRNA (fold)
ISG15
ISG15 mRNA (fold)
IFIT1
IFIT1 mRNA (fold)
NDR1(+/+)
NDR1(-/-)
c
IFN4α
IFN4α mRNA (fold)
VSV (h)
IFNβ
IFNβ mRNA (fold)
IL6
IL6 mRNA (fold)
IFNaR(+/+) siNC
IFNaR(+/+) siNDR1
IFNaR(-/-) siNC
IFNaR(-/-) siNDR1
ns
d
STAT1
(+/+)
(-/-)
siNC
siNDR1
STAT1
NDR1
GAPDH
e
NDR1
NDR1 mRNA (fold)
LM (h)
f
LM (h)
STAT1
NDR1
GAPDH
g
SOCS1
SOCS1 mRNA (fold)
IFNγ (h)
NOS2
NOS2 mRNA (fold)
CXCL9
CXCL9 mRNA (fold)
NDR(+/+)
NDR(-/-)
h
SOCS1
SOCS1 mRNA (fold)
LM (h)
NOS2
NOS2 mRNA (fold)
CXCL9
CXCL9 mRNA (fold)
NDR1(+/+)
NDR1(-/-)
i
Survival(%)
* p=0.0100
NDR1(+/+)
NDR1(-/-)
Days after LM infection

Supplementary Figure 5. NDR1 positively regulates interferon signaling pathways.

(**a**,**b**) Real-time PCR analysis of ISGs mRNA in RAW264.7 cells stably overexpressing NDR1 or its kinase-inactive mutants (**a**) or in wild-type and *NDR1*-deficient PMs (**b**), stimulated with IFNβ (100 IU ml^{-1}).

(**c**) Real-time PCR analysis of IFN4α, IFNβ and IL6 mRNA in wild-type and *IFNαR*-deficient BMDMs transfected with NDR1-specific or scrambled siRNA for 48 h, followed by infection with VSV.

(**d**) Immunoblot analysis of STAT1 expression in immortalized wild-type or *STAT1*-deficient BMDMs, treated with NDR1-specific or scrambled siRNA for 48 h.

(**e**,**f**) Real-time PCR (**e**) and Immunoblot analysis (**f**) of NDR1 expression in PMs infected with LM.

(**g**) Real-time PCR analysis of SOCS1, NOS2 and CXCL9 mRNA in wild-type and *NDR1*-deficient PMs treated with IFNγ (100 IU ml^{-1}).

(h) Real-time PCR analysis of ISGs mRNA in wild type and NDR1−/− PMs, infected with LM.

(i) Survival curve of 8-week-old male NDR1+/+ and NDR1-/- mice intravenously infected with LM (5×105CFU per mouse) (n=8 per group; Wilcoxon test). All mice were housed in SPF conditions.

Data are mean ± SD and are representative of three independent experiments. Student's t-test was used for statistical calculation. *$p<0.05$, **$p<0.01$, ***$p < 0.001$.

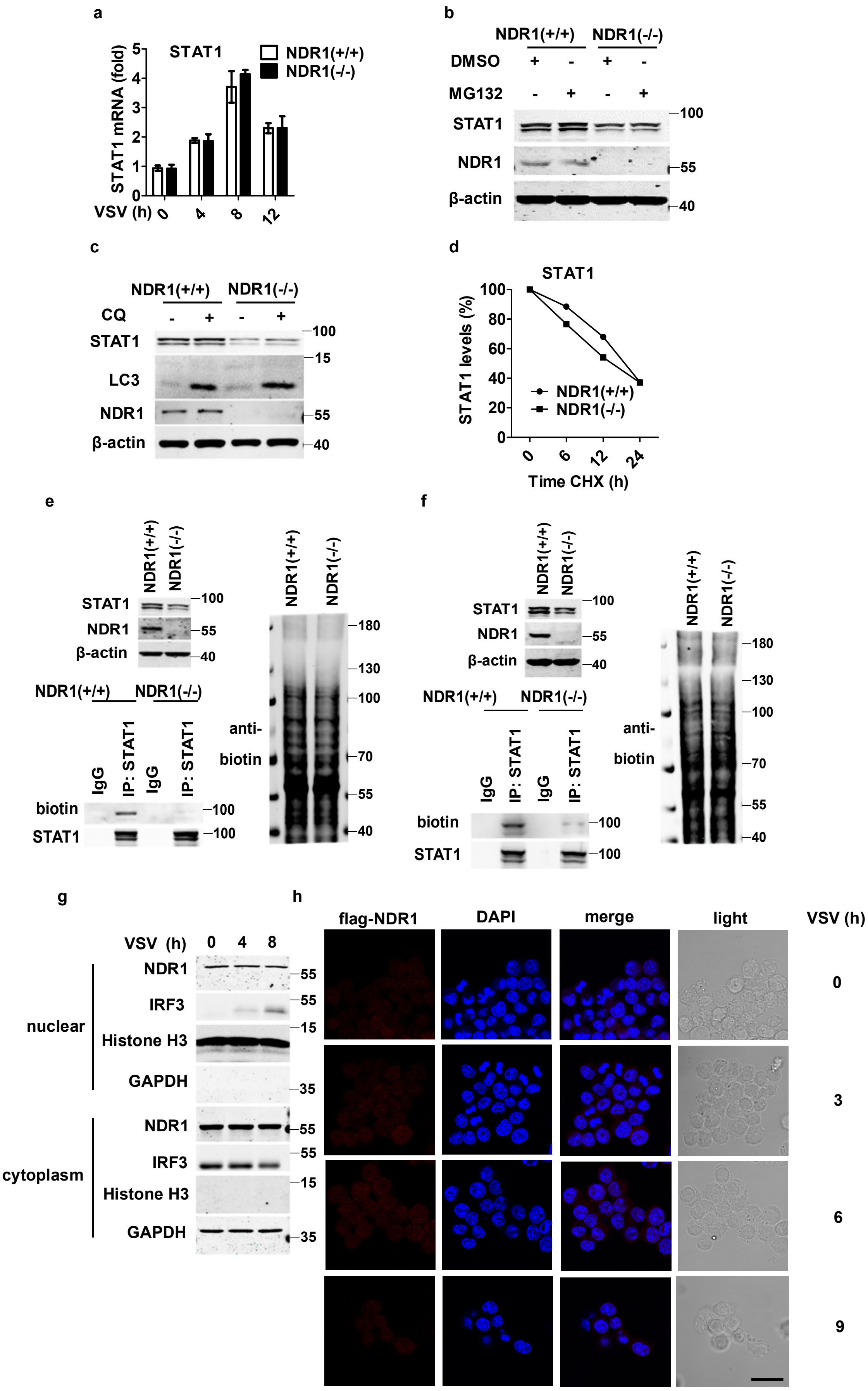
a
STAT1
NDR1(+/+)
NDR1(-/-)
STAT1 mRNA (fold)
VSV (h)
0
4
8
12
b
NDR1(+/+)
NDR1(-/-)
DMSO
MG132
STAT1
NDR1
β-actin
100
55
40
c
CQ
LC3
15
d
STAT1 levels (%)
Time CHX (h)
0
6
12
24
e
IgG
IP: STAT1
biotin
anti-biotin
180
130
70
f
g
VSV (h)
nuclear
cytoplasm
IRF3
Histone H3
GAPDH
35
h
flag-NDR1
DAPI
merge
light
3
6
9

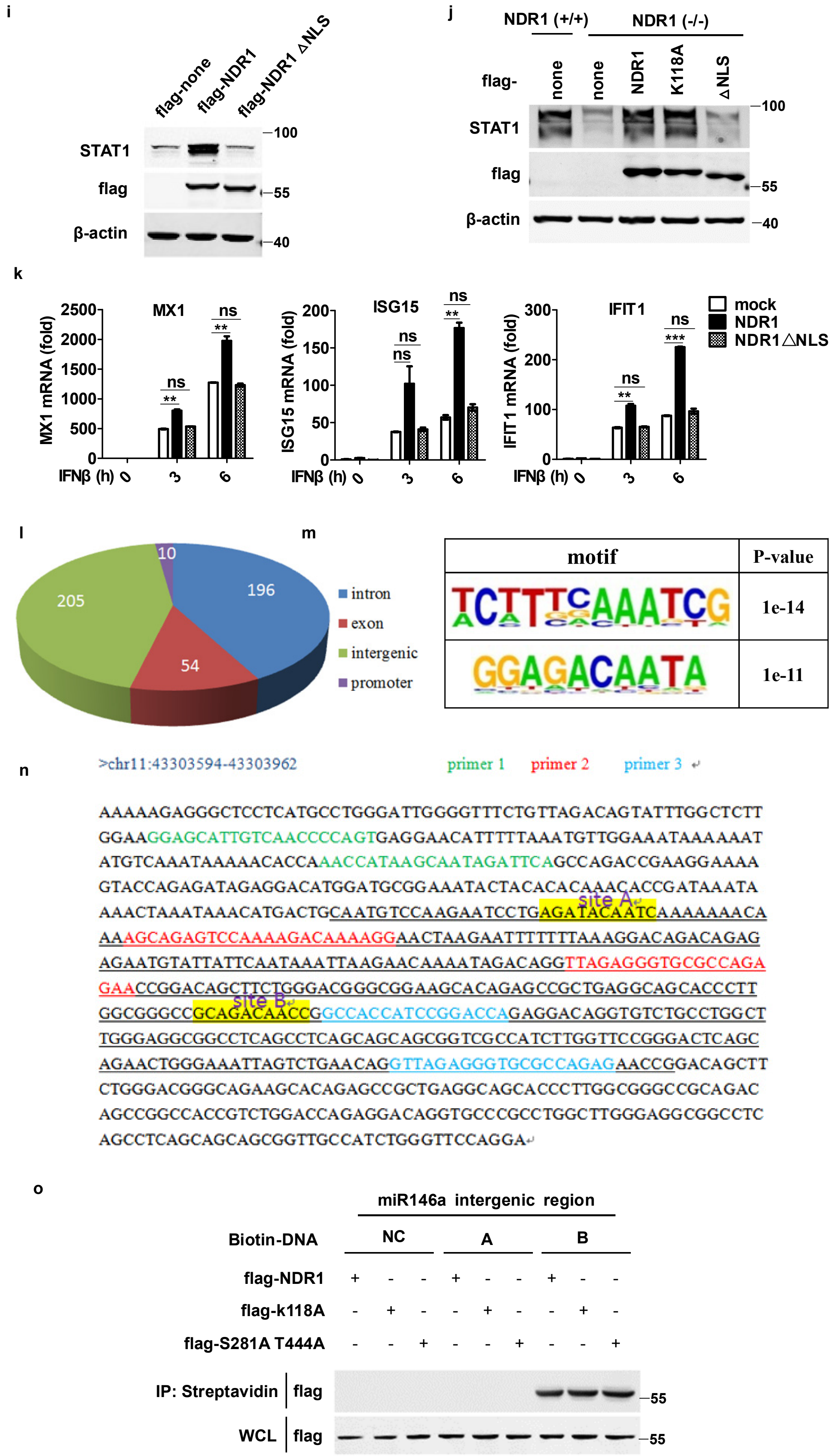

i
flag-none
flag-NDR1
flag-NDR1 △NLS
STAT1
flag
β-actin
100
55
40
j
NDR1 (+/+)
NDR1 (-/-)
flag-
none
none
NDR1
K118A
△NLS
STAT1
flag
β-actin
100
55
40
k
MX1
MX1 mRNA (fold)
ISG15
ISG15 mRNA (fold)
IFIT1
IFIT1 mRNA (fold)
IFNβ (h)
0
3
6
ns
**
***
mock
NDR1
NDR1△NLS
l
10
196
205
54
intron
exon
intergenic
promoter
m
motif
P-value
TCTTTCAAATCG
1e-14
GGAGACAATA
1e-11
n
>chr11:43303594-43303962
primer 1
primer 2
primer 3
AAAAAGAGGGCTCCTCATGCCTGGGATTGGGGTTTCTGTTAGACAGTATTTGGCTCTT
GGAAGGAGCATTGTCAACCCCAGTGAGGAACATTTTTAAATGTTGGAAATAAAAAAT
ATGTCAAATAAAAACACCAAACCATAAGCAATAGATTCAGCCAGACCGAAGGAAAA
GTACCAGAGATAGAGGACATGGATGCGGAAATACTACACACAAACACCGATAAATA
AAACTAAATAAACATGACTGCAATGTCCAAGAATCCTGAGATACAATCAAAAAAACA
AAAGCAGAGTCCAAAAGACAAAAGGAACTAAGAATTTTTTTAAAGGACAGACAGAG
AGAATGTATTATTCAATAAATTAAGAACAAAATAGACAGGTTAGAGGGTGCGCCAGA
GAACCGGACAGCTTCTGGGACGGGCGGAAGCACAGAGCCGCTGAGGCAGCACCCTT
GGCGGGCCGCAGACAACCGGCCACCATCCGGACCAGAGGACAGGTGTCTGCCTGGCT
TGGGAGGCGGCCTCAGCCTCAGCAGCAGCGGTCGCCATCTTGGTTCCGGGACTCAGC
AGAACTGGGAAATTAGTCTGAACAGGTTAGAGGGTGCGCCAGAGAACCGGACAGCTT
CTGGGACGGGCAGAAGCACAGAGCCGCTGAGGCAGCACCCTTGGCGGGCCGCAGAC
AGCCGGCCACCGTCTGGACCAGAGGACAGGTGCCCGCCTGGCTTGGGAGGCGGCCTC
AGCCTCAGCAGCAGCGGTTGCCATCTGGGTTCCAGGA
site A
site B
o
miR146a intergenic region
Biotin-DNA
NC
A
B
flag-NDR1
flag-k118A
flag-S281A T444A
IP: Streptavidin
WCL
flag
55

Supplementary Figure 6. NDR1 specifically promotes STAT1 translation via binding to the intergenic region of miR146a to act as a transcriptional regulator.

(**a**) Real-time PCR analysis of STAT1 mRNA in wild-type and *NDR1*-deficient PMs infected with VSV.

(**b**,**c**) Immunoblot analysis of STAT1 expression in lysates of wild-type and *NDR1*-deficient PMs, treated with MG132 (10 μM) (**b**) or with CQ (100 μM) (**c**).

(**d**) Wild-type and *NDR1*-deficient PMs were treated with cycloheximide (75 μg ml^{-1}) and harvested for immunoblot analysis. STAT1 densitometry was presented relative to β-actin. And relative STAT1 quantification levels were normalized with the STAT1 densitometry on the resting state.

(**e,f**) Wild-type and *NDR1*-deficient BMDMs (**e**) or HEK293 cells (**f**) were treated with metabolic labeling reagents (AHA). Cell lysates were labeled with biotin for nascent proteins via azide/alkyne reaction, followed by immunoprecipition with anti-STAT1 antibody or nonspecific IgG.

(**g**) Immunoblot analysis of NDR1 expression in cytoplasmic and nuclear fractions from PMs infected with VSV.

(**h**) Confocal microscopy of RAW264.7 stably overexpressing flag-NDR1. Cells were stained with Alexa Fluor 555–conjugated antibody to flag (red). DAPI serves as a marker of nuclei (blue). Scale bar, 20 μm.

(**i**) Immunoblot analysis of STAT1 expression in HEK293 cells transfected with an empty vector or vectors expressing full-length NDR1 or NDR1 △NLS mutant.

(**j**) Immunoblot analysis of STAT1 expression in wild-type and NDR1-deficient PMs transfected with an empty vector, or vectors encoding NDR1 or its kinase-inactive mutants.

(**k**) Real-time PCR analysis of ISGs expression in RAW264.7 cells stably expressing NDR1, NDR1 △NLS mutant or empty vector, treated with IFNβ (100 IU ml^{-1}).

(**l**) Distribution of NDR1 ChIP-seq peaks among various gene regions (key) in RAW264.7 cells stably expressing flag-NDR1.

(**m**) NDR1-binding motif identified by analysis of NDR1 ChIP-seq peaks in flag-NDR1 stable RAW264.7 cells. left, letter size indicates frequency of nucleotide. Right, significance of motif occurrence.

(**n**) The sequence of murine miR146a intergenic region. The sequence of NDR1-binding peak in miR146a intergenic region was underlined; the putative NDR1 binding sites were highlighted. The sequence of the primers for multisite ChIP-qPCR assay was marked in green, red and blue respectively.

(**o**) Biotin-labeled DNA (miR146a intergenic region or control DNA) was incubated with lysates of HEK293 cells transfected with NDR1 or its kinase-inactive mutants vectors and precipitated with streptavidin sepharose beads. The beads were washed and then resolved by 2× SDS loading buffer for immunoblotting. Data are mean ± SD and are representative of three independent experiments. Student's t-test was used for statistical calculation. $^*p<0.05$, $^{**}p<0.01$, $^{***}p < 0.001$.

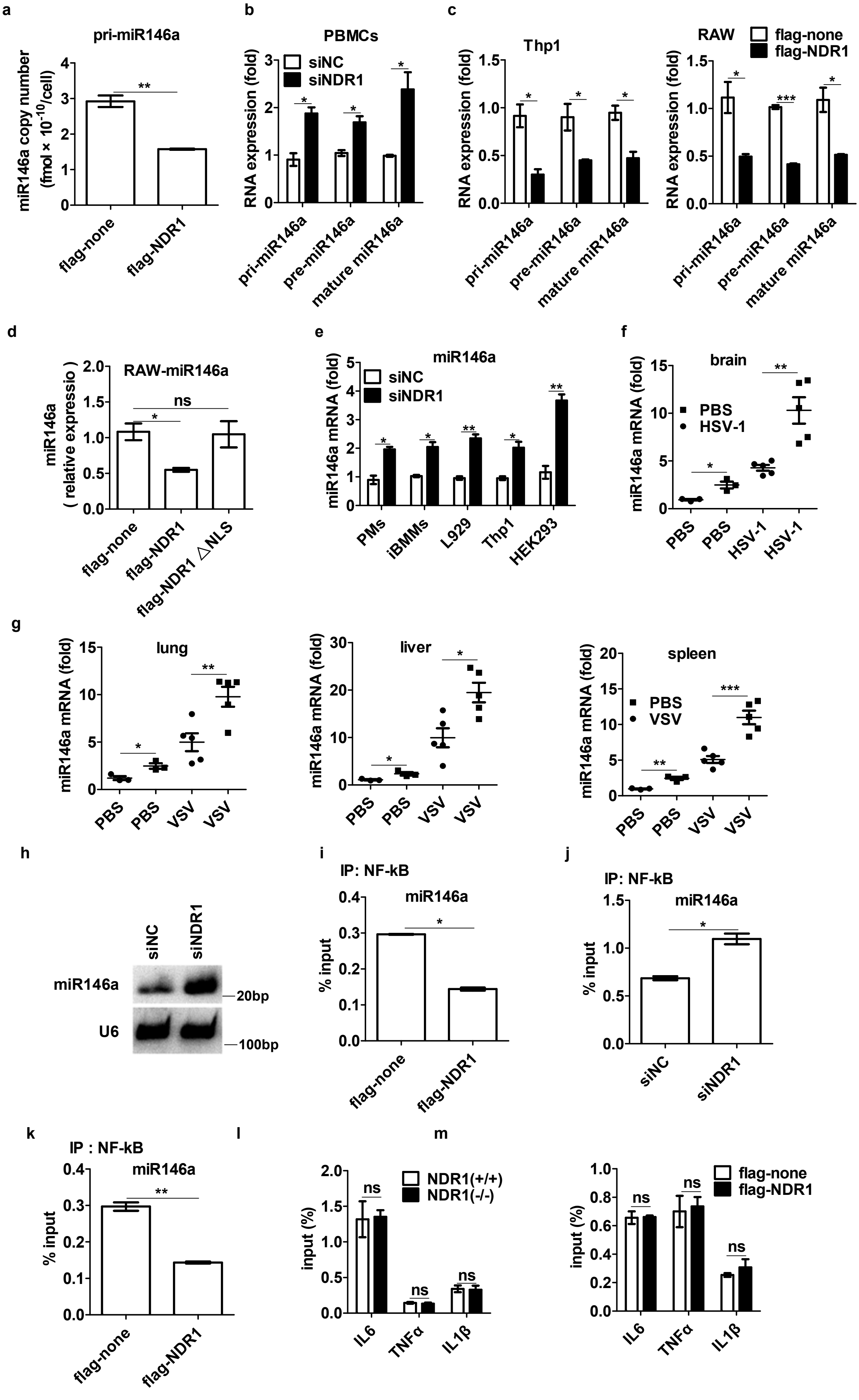
a
pri-miR146a
miR146a copy number (fmol × 10-10/cell)
flag-none
flag-NDR1
b
PBMCs
siNC
siNDR1
RNA expression (fold)
pri-miR146a
pre-miR146a
mature miR146a
c
Thp1
RAW
flag-none
flag-NDR1
d
RAW-miR146a
miR146a (relative expressio)
ns
flag-NDR1 △NLS
e
miR146a
miR146a mRNA (fold)
PMs
iBMMs
L929
Thp1
HEK293
f
brain
PBS
HSV-1
g
lung
liver
spleen
VSV
h
miR146a
U6
20bp
100bp
i
IP: NF-kB
% input
j
k
IP : NF-kB
l
NDR1(+/+)
NDR1(-/-)
input (%)
IL6
TNFα
IL1β
m

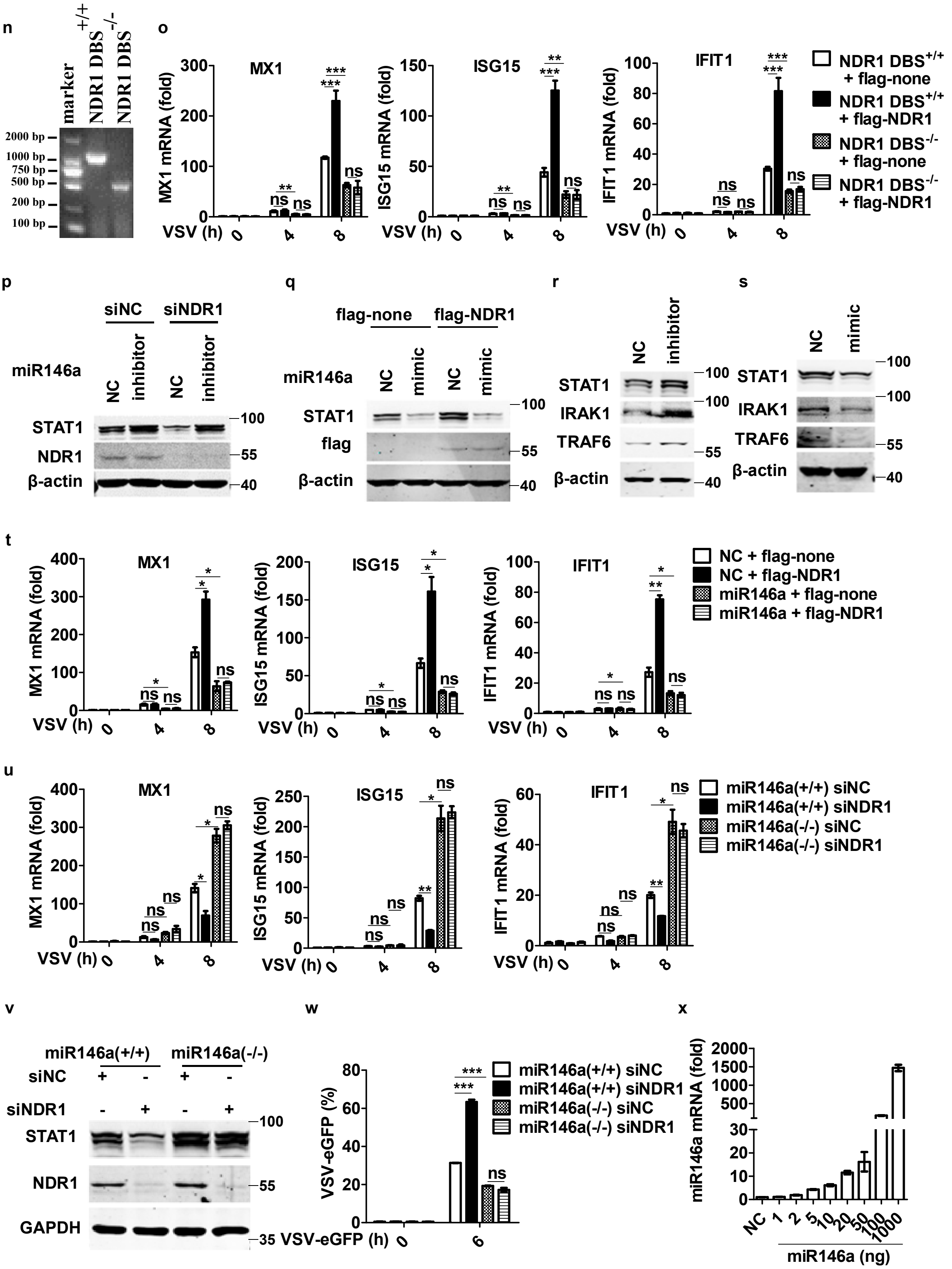
n
o
p
q
r
s
t
u
v
w
x
marker
NDR1 DBS+/+
NDR1 DBS-/-
2000 bp
1000 bp
750 bp
500 bp
200 bp
100 bp
MX1
ISG15
IFIT1
MX1 mRNA (fold)
ISG15 mRNA (fold)
IFIT1 mRNA (fold)
VSV (h)
NDR1 DBS+/+ + flag-none
NDR1 DBS+/+ + flag-NDR1
NDR1 DBS-/- + flag-none
NDR1 DBS-/- + flag-NDR1
siNC
siNDR1
miR146a
NC
inhibitor
STAT1
NDR1
β-actin
flag-none
flag-NDR1
mimic
flag
IRAK1
TRAF6
NC + flag-none
NC + flag-NDR1
miR146a + flag-none
miR146a + flag-NDR1
miR146a(+/+) siNC
miR146a(+/+) siNDR1
miR146a(-/-) siNC
miR146a(-/-) siNDR1
miR146a(+/+)
miR146a(-/-)
GAPDH
VSV-eGFP (%)
VSV-eGFP (h)
miR146a mRNA (fold)
miR146a (ng)

Supplementary Figure 7. NDR1 promotes STAT1 translation via targeting miR146a.

(**a**) Absolute quantification PCR analysis of pri-miR146a expression in RAW264.7 cells stably overexpressing NDR1 or empty vector. Serial 10-fold dilutions from mouse pre-miR146a overexpressing plasmid were subjected to real-time PCR analysis by pri-miR146a primers. The standard curve of pri-miR146a was generated according to averaged fluorescence values and the corresponding amounts of the plasmid. The amounts of pri-miR146a in per cell were calculated according to the standard curve.

(**b,c**) Real-time PCR analysis of pri-, pre- and mature miR146a expression in human PBMCs treated with NDR1-specific or scrambled siRNA (**b**) or in Thp1 and RAW264.7 cells stably overexpressing NDR1 or empty vector (**c**). TaqMan microRNA assay using specific hydrolysis probes for mature miR146a and U6 was performed to achieve specificity.

(**d**) Real-time PCR analysis of mature miR146a expression in RAW264.7 cells stably overexpressing NDR1, NDR1 △NLS mutant or empty vector using specific hydrolysis probes.

(**e**) Real-time PCR analysis of mature miR146a expression in PMs, immortalized BMDMs, L929, human PBMCs or HEK293 cells treated with NDR1-specific or scrambled siRNA.

(**f,g**) Real-time PCR analysis of mature miR146a expression in brains from *NDR1*$^{+/+}$ and *NDR1*$^{-/-}$ mice intravenously infected with or without HSV (1×10^6 pfu per mouse) for 4 days (**f**), or in lung, liver and spleen of *NDR1*$^{+/+}$ and *NDR1*$^{-/-}$ mice intraperitoneally injected with PBS or VSV (1×10^7 pfu g^{-1}) for 12 h (**g**).

(**h**) Northern blot analysis of mature miR146a expression in Thp1 cells treated with NDR1-specific or scramble siRNA.

(**i,j**) ChIP-qPCR analysis of the enrichment of NF-κB to miR146a promoter with anti-NF-κB

antibody in lysates of Thp1 cells stably overexpressing NDR1 or empty vector (**i**) or treated with NDR1-specific or scrambled siRNA (**j**). MiR146a promoter sequences in input DNA and DNA recovered from antibody-bind chromatin segments were detected by qPCR. IgG serves as negative control and input serves as normalized control.

(**k**) ChIP-qPCR analysis of the enrichment of NF-κB to murine miR146a regulatory sequences with anti-NF-κB antibody in lysates of RAW264.7 cells stably overexpressing NDR1 or empty vector. MiR146a regulatory sequences in input DNA and DNA recovered from antibody-bind chromatin segments were detected by qPCR. IgG serves as negative control and input serves as normalized control.

(**l**,**m**) ChIP-qPCR analysis of the enrichment of NF-κB to IL6, TNFα and IL1β promoters with anti-NF-κB antibody in lysates of wild-type or *NDR1*-deficient PMs (**l**) or of RAW264.7 cells stably expressing NDR1 or control transfectants (**m**). IL6, TNFα and IL1β promoter sequences in input DNA and DNA recovered from antibody-bind chromatin segments were detected by qPCR. IgG serves as negative control and input as normalized control.

(**n**) PCR analysis of genomic DNA from NDR1 DNA binding site deficient (NDR1 $DBS^{-/-}$) and control (NDR1 $DBS^{+/+}$) L929 cells for genotype identification.

(**o**) Real-time PCR analysis of ISGs induction in NDR1 $DBS^{+/+}$ and NDR1 $DBS^{-/-}$ L929 cells transfected with flag-NDR1 or empty vector and followed by VSV infection for the indicated times.

(**p**) Immunoblot analysis of STAT1 expression in PMs treated with NDR1-specific or control siRNA for 36 h, followed by transfection of miR146a inhibitor or negative control.

(**q**) Immunoblot analysis of STAT1 in RAW264.7 cells stably overexpressing NDR1 or control transfectants, transfected with miR146a mimic or negative control.

(**r**) Immunoblot analysis of STAT1, IRAK1 and TRAF6 expression in PMs transfected with miR146a inhibitor or negative control for 36 h.

(**s**) Immunoblot analysis of STAT1, IRAK1 and TRAF6 expression in RAW264.7 cells transfected with miR146a mimics or negative control for 36 h.

(**t**) Real-time PCR analysis of ISGs induction in L929 cells stably overexpressing pre-miR146a or empty vector, transfected with vector expressing flag-NDR1 or flag-none and followed by VSV infection for the indicated times.

(**u**) Real-time PCR analysis of ISGs induction in WT and *miR146a*$^{-/-}$ L929 cells treated NDR1-specific or scrambled siRNA for 48 h, followed by VSV-eGFP infection.

(**v**) Immunoblot analysis of STAT1 in lysates of wild-type and miR146a-deficient HEK293 cells, treated with NDR1-specific or scrambled siRNA for 48 h.

(**w**) FACS analysis of wild-type and miR146a-deficient HEK293 cells treated as in (**r**), followed by infection of VSV-eGFP.

(**x**) 2 × 10^5 HEK293 cells were transfected with empty vector or increasing concentrations of vectors for the miR146a constructs. After 48 h, cells were used for Real-time PCR analysis of mature miR146a expression. Data are mean ± SD and are representative of three independent experiments. Student's t-test was used for statistical calculation. *$p<0.05$, **$p<0.01$, ***$p < 0.001$.

a

b

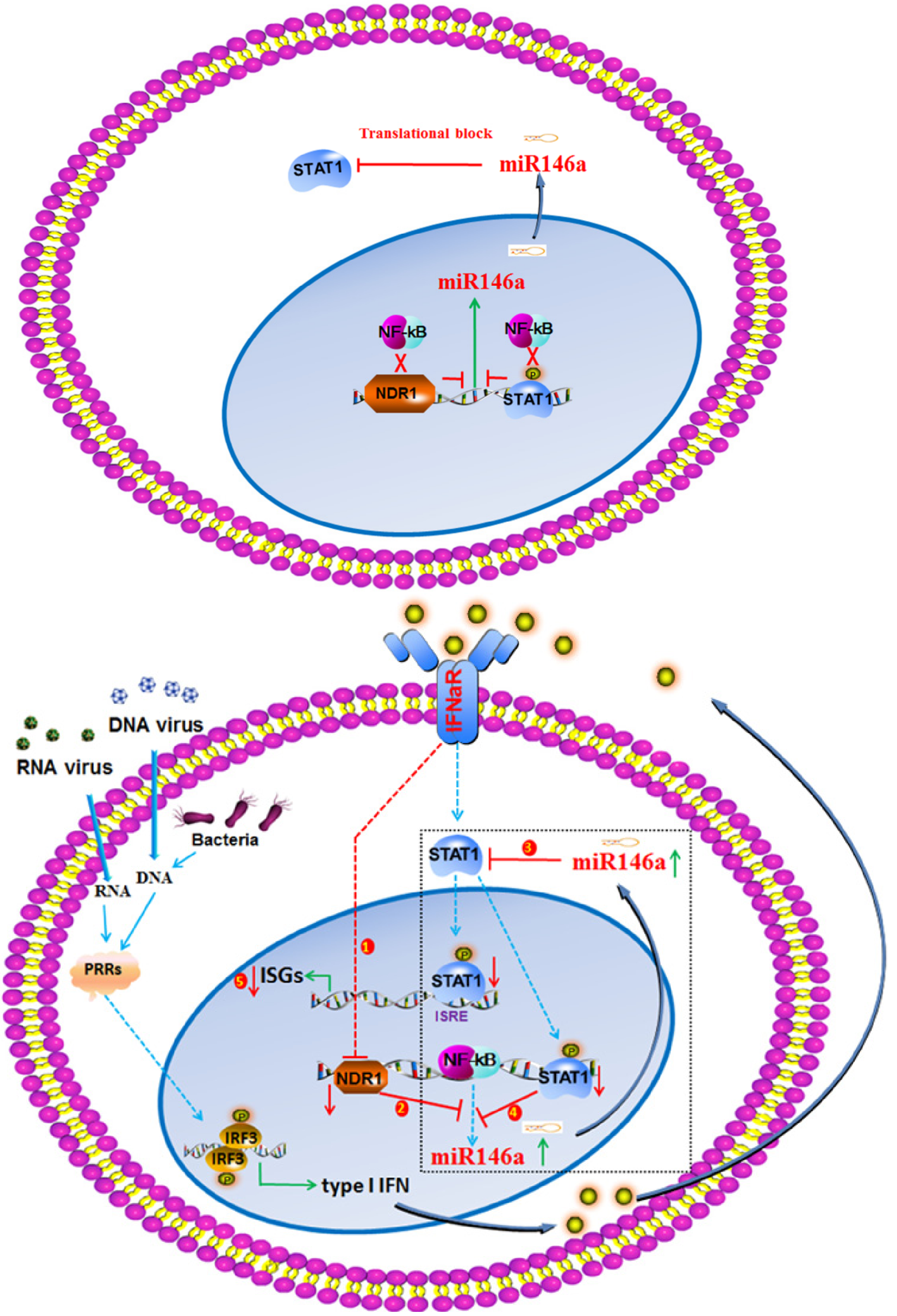

Supplementary Figure 8. Downregulation of NDR1 due to viral infection inhibits the innate immune response by initiating an miR146a-STAT1 feedback loop.

(**a**) NDR1 and STAT1 act as trans-repressors to inhibit NF-κB-mediated miR146a transcription, which

keeps miR146a at low transcriptional level at the resting state.

(**b**) Upon pathogenic microorganism invasions, NDR1 expression is down-regulated by type I IFN pathway[1]; Reduced NDR1 facilitates NF-κB-mediated miR146a transcription[2]; Up-regulated miR146a leads to the inhibition of STAT1 translation and pathogenic infection-induced STAT1 phosphorylation[3]; Decreased STAT1 promotes miR146a expression via the feedback loop between STAT1 and miR146a[4], which ultimately dampens ISGs induction and benefits pathogens to evade innate immune response[5].

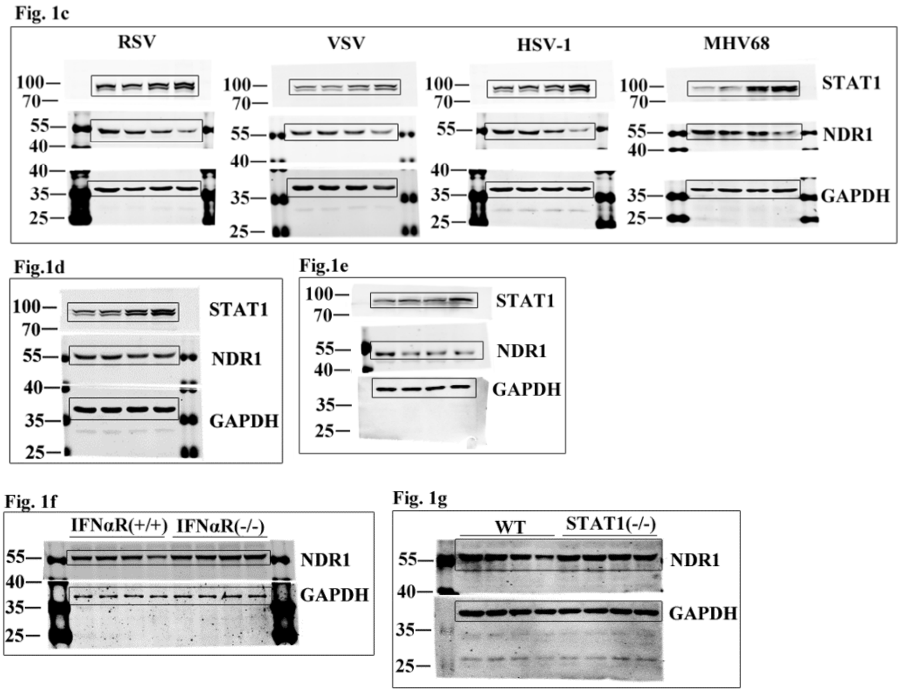

Supplementary Figure 9. Uncropped scans of the western blots related to Figure 1

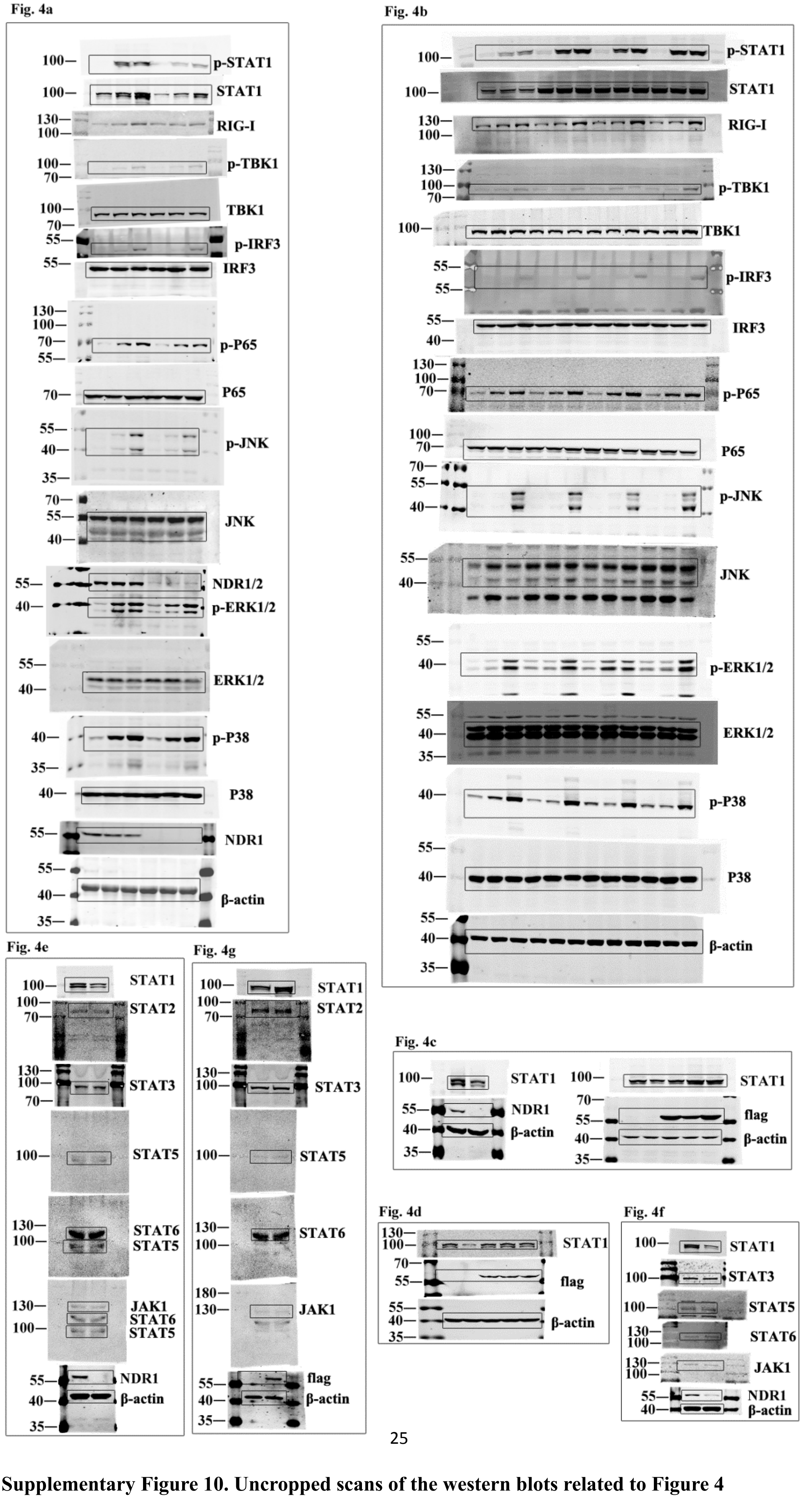

Supplementary Figure 10. Uncropped scans of the western blots related to Figure 4

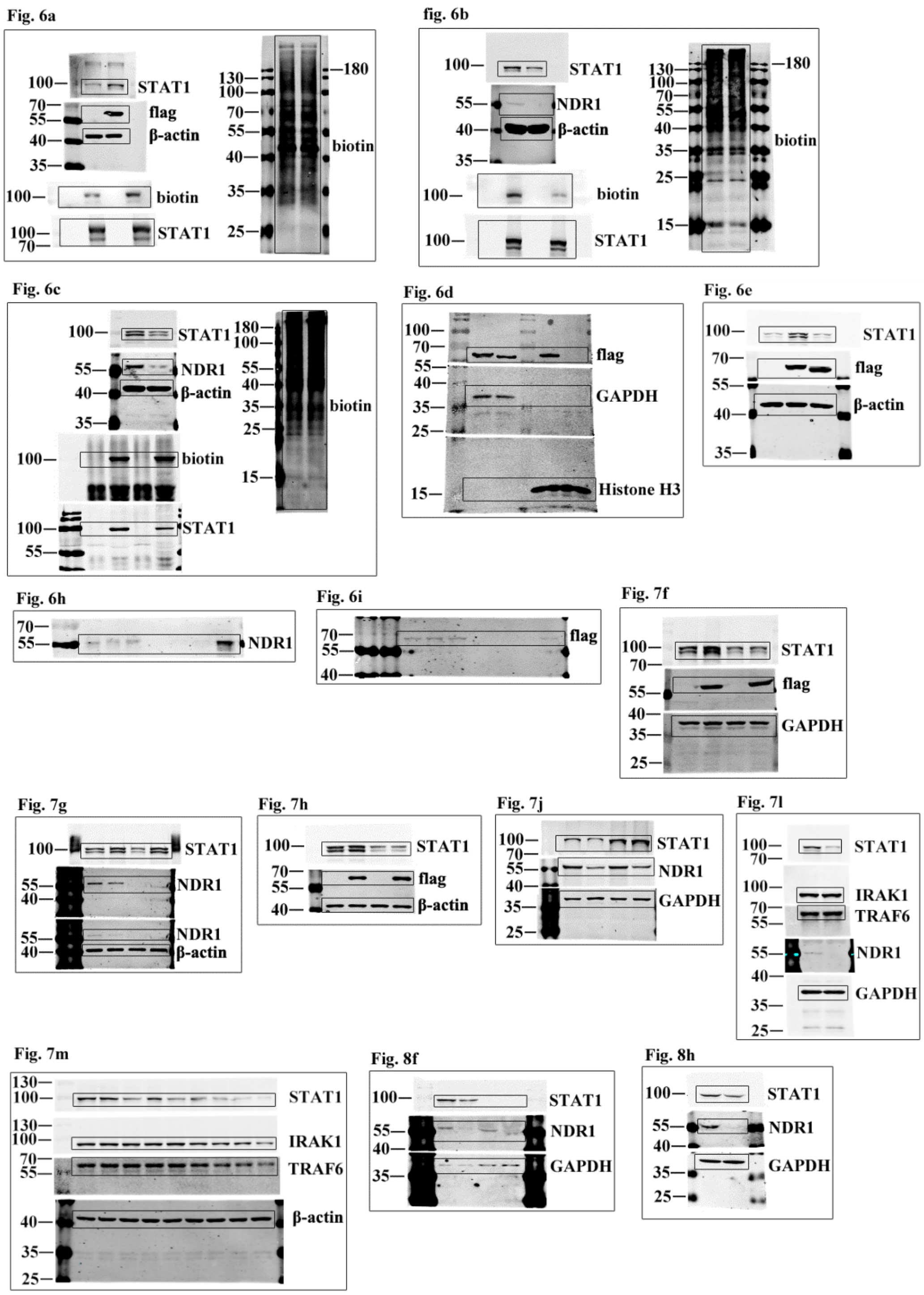

Supplementary Figure 11. Uncropped scans of the western blots related to Figure 6-8

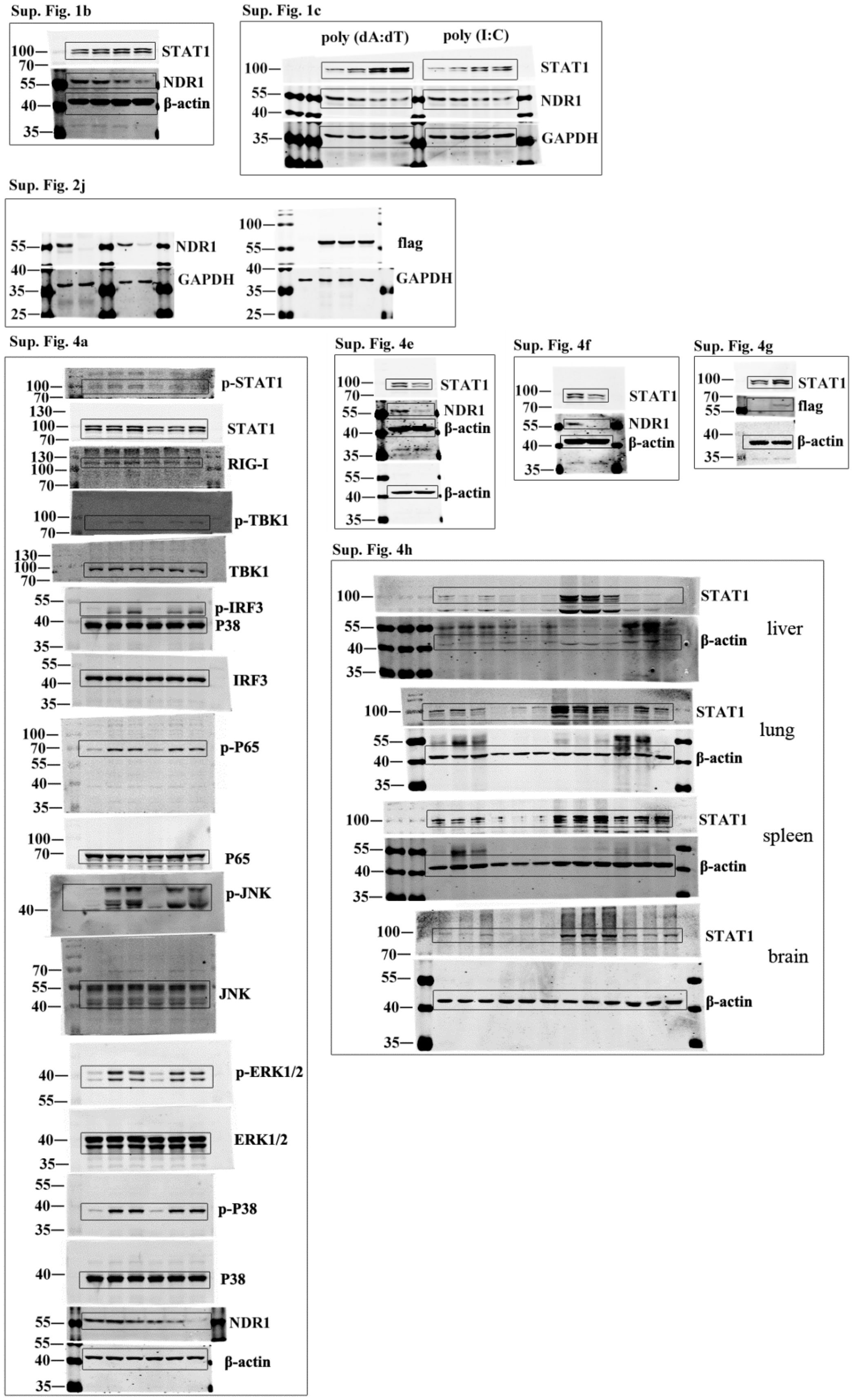

Supplementary Figure 12. Uncropped scans of the western blots related to Supplementary Figure 1-4

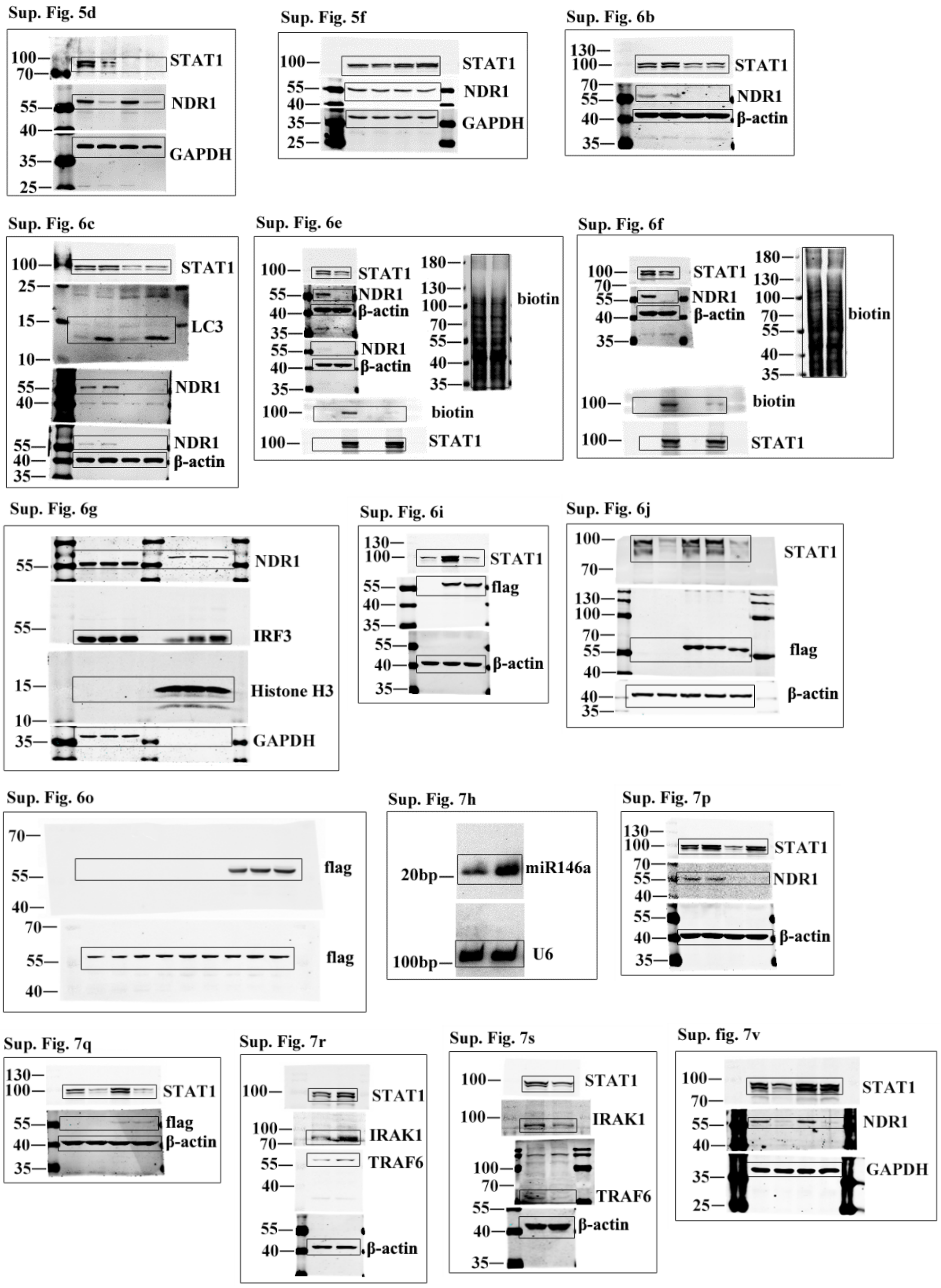

Supplementary Figure 13. Uncropped scans of the western blots related to Supplementary Figure 5-7

Supplementary Methods

Viral and bacterial infections

Cells were infected with VSV (kindly provided by Dr. Huazhang An, Second Military Medical University, Shanghai, multiplicity of infection (MOI) =0.1), VSV-GFP (kindly provided by Dr. Zongping Xia, Life Science Institute, Zhejiang University, Hangzhou, MOI=1), H1N1 (Influenza A virus PR8/A/34, kindly provided Dr. Jing Qian, Zhejiang University School of Medicine, Hangzhou, MOI=1), RSV (subtype A, strain Long, kindly provided Dr. Jing Qian, MOI=1), MHV68 (kindly provided Dr. Jing Qian, MOI=5), HSV-1 (kindly provided by Dr. Ping Wang, Shanghai 10th People's Hospital, Department of Central Laboratory, Shanghai, MOI=5), HSV-eGFP (kindly provided by Dr. Pinglong Xu, Life Science Institute, Zhejiang University, Hangzhou, MOI=5), HCV JFH-1 (kindly provided by Dr. Xiaoben Pan, Peking University Hepatology Institute, Beijing, MOI=1), and listeria monocytogenes (kindly provided Dr. Lie Wang, Zhejiang University School of Medicine, Hangzhou, MOI=10) for the indicated times. Cytokine production was analyzed at the indicated times. For in vivo studies, 8-weeks-old male groups of littermate mice were infected with VSV (1×10^7 pfu g^{-1}, i.p., 12 h, n=6 per group) for the determination of IFNβ, IL6 and TNFα production in serum, PMs and organs, and the determination of viral titer in organs. For *in vivo* survival studies, 8-weeks-old male groups of littermate mice were infected intravenously with VSV (1×10^8 pfu g^{-1}, i.v., n=9 per group). For HSV infection *in vivo*, HSV (1×10^6 pfu per mouse, i.v., n=10 per group) was injected intravenously into 8-weeks-old male mice for the determination of IFNβ, IL6 and TNFα production in serum (6 h) and viral load in brain (4 days). For L. monocytogenes infection studies, 8-weeks-old male wild-type and *NDR1*-deficient littermate mice were administered intravenously with listeria monocytogenes (5×10^5 c.f.u. per mouse, n=8 per group), and were monitored for 7 days for morbidity and mortality.

Sequences, plasmids and transfection

Mutant expression plasmids for NDR1 (K118A, S281A T444A, ∆NLS) were generated using the KOD-Plus-Neo kit (Toyobo). Mmu-pre-miR-146a in CD513 construct was purchase from System Biosciences (California, USA). All constructs were confirmed by DNA sequencing. For mouse PMs, INTERFERin (Ployplus) was used for siRNAs transfection, and plasmids were transfected into cells with lipo3000 (Invitrogen) according to the manufacturer's protocol. For RAW264.7 stable cell lines, plasmids were transfected into cells with jetPRIME reagent (Ployplus) according to the manufacturer's protocol, then puromycin (8 μg ml^{-1}) (Sigma-Aldrich) was used for selection and single-cell colonies cells were picked and validated at protein level by western blot. For Thp1 stable cell lines, Thp1 cells were infected with flag-NDR1 or flag-none overexpression lentivirus and selected with puromycin (4 μg ml^{-1}). Chemically synthesized siRNA duplexes were obtained from Gene-Pharma. The siRNA duplexes were transfected into macrophages, L929 and HEK293 cells with INTERFERin reagent (Polyplus) according to a standard protocol. The sequences of siRNA used in this study are as follows:

Control siRNA: UUCUCCGAACGUGUCACGU

siNDR1 (mouse): GCUAAACCUCUACCUAAUCAUTT

siNDR1 (human): GCACAUGCUCGGAAGGAAATT

siSTAT1 (human): GACCCAAUCCAGAUGUCUATT

Generation and validation of NDR1, NDR1 DBS and miR146a-KO Cells

The NDR1, miR146a and NDR1 DBS knockout (KO) cells were constructed using the CRISPR/Cas9 gene-editing system. The CRISPR plasmid pEP-330x (Addgene) contains expression cassettes of Cas9 and puromycin resistant gene. The target sequences of gRNAs were designed using the MIT online tool (http://crispr.mit.edu/). To generate NDR1-KO cells, two gRNAs targeting the exons of NDR1 were

designed and inserted into the pEP-330x vector by using the BpiI (Thermo) site, and then co-transfected into HEK293 cells using PEI (Polysciences, cat# 23966-2) for 48 h; To generate NDR1 DBS KO cells, we designed two gRNAs to target the upstream and downstream genomic sequence of the DNA binding site of NDR1 in miR146a intergenic region and cloned into the pEP-330x vector, then the plasmids were co-transfected into L929 cells using PEI for 48 h; To generate miR146a-KO cells, two gRNAs targeting the upstream and downstream flanking genomic sequence of pre-miR146a were designed and inserted into the pEP-330x vector, and then co-transfected into HEK293 or L929 cells using PEI for 48 h. Then puromycin (Sigma-Aldrich) was used for selection in HEK293 (2 μg ml^{-1}), L929 (8 μg ml^{-1}). To validate and pick out the knockout cell colonies, genomic DNA was extracted from the parental and knockout cells, and validated at DNA level by PCR. Primers for constructing CRISPR-CAS9 plasmids and genotype identification are listed in **Supplementary Table 4**.

NDR1-WT and –KO J2 BMDMs immortalization

The AMJ2-C11 cell line was gift from Dr. Genhong Cheng (University of California, Los Angeles), cultured in RPMI-1640 medium supplemented with 10% heat-inactivated FBS, 10mM Hepes (PH, 7.8), 100U ml^{-1} penicillin and 100μg ml^{-1} streptomycin, and used for J2 recombinant retrovirus preparation[1, 2]. The supernatant containing J2 viruses was harvested and filtered through 0.22 μm filter. NDR1-WT and –KO bone marrow cells were infected with the J2 retrovirus and immortalized as J2 BMDMs[3].

ELISA

Cell culture supernatants and sera were assayed by ELISA for mouse IL6, TNFα (eBioscience) and IFN-β (Biolegend), according to the manufacturers' instructions.

RNA isolation, relative and absolute real-time quantitative PCR

Total RNA was extracted using TRIzol reagent (Life Technologies) according to the manufacturer's

instructions. The miR146a precursor and primary transcript were reverse-transcribed by random N6 primers (Thermo) using HiScript II Q Select RT SuperMix (Vazyme) according to the following procedure: 0.5 μg total RNA, 0.5 μL Random Hexamer Primer (100 μM) and 4 × gDNA wiper Mix were incubated at 42°C for 2min to remove genomic DNA contamination, then 5 × HiScript II Select qRT SuperMix II was added to the reaction mixture and incubated at 50°C for 15min and 85°C for 2min. The relative real-time quantitative RT-PCR (qRT-PCR) analysis was performed using SYBR Green master mix (Vazyme) on a CFX-Touch system (Bio-Rad) and the U6 small nuclear RNA was used as an internal control. The PCR primers for pri-, pre-miR146a and U6 are listed in **Supplementary Table 5**.

The expression of mature miR146a was evaluated using two qRT-PCR systems. One is TaqMan MicroRNA Assays: the specific primers for mature miR146a (Applied Biosystems) were used for reverse transcription with TaqMan MicroRNA Reverse Transcription Kit (Applied Biosystems), and the qRT-PCR analysis was performed using miR146a specific PCR primers and TaqMan MGB probe (Applied Biosystems) with Premix EX Taq kit (Takara) on a Roche 480 real-time PCR system (Roche)[4]. Another one was performed as follows: the specific primers for mature miR146a (5’-GTCGTATCCAGTGCAGGGTCCGAGGTATTCGCACTGGATACGACAACCCA-3’) and U6 (5’-AACGCTTCACGAATTTGCGT-3’) were used for reverse transcription; the qRT-PCR analysis was performed using SYBR Green master mix (Vazyme) on a CFX-Touch system (Bio-Rad). The PCR primers for mature miR146a and U6 are listed in **Supplementary Table 5.** The relative expression level of miR146a was normalized to that of internal control U6 by using the $2^{-\Delta\Delta Ct}$ method.

For absolute real-time quantitative PCR analysis of pri-miR146a expression, 1×10^6 cells were harvested for the extraction of total RNA, which was dissolved in 10 μL DEPC treated water. Then we

took 2μL RNA solution of each sample for reverse transcription and diluted cDNA solution up to 100 μL. Finally, 4 μL cDNA solution was used for qRT-PCR using SYBR Green master mix (Vazyme) on a CFX-Touch system (Bio-Rad). Serial 10-fold dilutions from mouse pre-miR146a overexpressing plasmid were subjected to real-time PCR analysis by pri-miR146a primers. The standard curve of pri-miR146a was generated according to averaged fluorescence values and the corresponding amounts of the plasmid. The amounts of pri-miR146a in per cell were calculated according to the standard curve. The primer of mouse pri-miR146a for PCR was designed according to the upstream and downstream flanking genomic sequence of mouse pre-miR146a which was obtained from DNA sequencing.

For other mRNA analysis, RNA was reverse transcribed using M-MLV reverse transcriptase (Takara) and relative real-time quantitative RT-PCR (qRT-PCR) analysis was performed using SYBR Green master mix (Vazyme) on a CFX-Touch system (Bio-Rad). Data were normalized by the level of β-actin or GAPDH expression in each individual sample. For other mRNA analysis, the primers are listed in **Supplementary Table 5**.

Northern blot analysis

Briefly, 30 μg of total RNA per sample was resolved in 17% urea-acrylamide gels, transferred onto a positively charged nylon membrane (Ambion, Grand Island, NY) in 0.5 × TBE and immobilized by ultraviolet cross-linking and baking at 80°C for 1 h. The membranes were pre-hybridized at 37°C for 2 h and then hybridized at 37°C overnight to a digoxigenin (DIG)-labeled DNA oligonucleotide probe that was complementary to the sequence of mature miR-146a (5'-AACCCATGGAATTCAGTTCTCA) or to U6 small nuclear RNA (snRNA) (5'-GCAGGGGCCATGCTAATCTTCTCTGTATG-3')[5]. DIG Northern Starter Kit from Roche (Indianapolis, IN) Applied Science was used to visualize the signals[6].

Assay of luciferase activity

HEK293 cells were co-transfected with a mixture of the indicated firefly luciferase reporter plasmid, the pRL-TK-Renilla-luciferase reporter assay plasmid, and any appropriate additional constructs for 18~24 hours. Total amounts of DNA were equalized with empty control vector. Luciferase activity was measured with the Dual-Luciferase Reporter Assay system (Promega) according to the manufacturer's instructions. Data were normalized for transfection efficiency by division of firefly luciferase activity by that of Renilla luciferase.

Coimmunoprecipitation and immunoblot analysis

For immunoblot analysis, cells or tissues were lysed with cell lysis buffer (Cell Signaling Technology) supplemented with a protease inhibitor cocktail (Sigma-Aldrich). Protein concentrations in the extracts were measured with a BCA protein assay kit (Pierce) and were made equal with extraction reagent. For immunoprecipitation (IP), whole-cell extracts were collected 36 h after transfection and were lysed in IP buffer containing 1.0% (vol/vol) Triton X-100; 10% (vol/vol) glycerol; 100 mM Tris-HCl, pH 7.4; 1 mM EDTA; 75 mM NaCl; and a protease inhibitor cocktail (Sigma-Aldrich). After centrifugation for 10 min at 12,000 × g at 4°C， supernatants were collected and incubated with protein G magnetic beads (Bio-Rad) together with 1 μg corresponding antibodies. After 4h of incubation, beads were washed three times with IP buffer. Immunoprecipitates were eluted by boiling with 1% (wt/vol) SDS sample buffer. For immunoblot analysis, immunoprecipitates or whole-cell lysates were loaded and subjected to SDS–PAGE, transferred onto nitrocellulose membranes and then blotted with indicated antibodies which are list in **Supplementary Table 1**.

Labeling of newly synthesized proteins with biotin

Cells were depleted of their methionine reserves through a 1 hour incubation in methionine-free

medium (DMEM non-GMP formulation without methionine, arginine, and lysine; Invitrogen) containing 10% dialyzed FBS (Invitrogen), 80 μM L-glutamine (Invitrogen), and 200 μM L-cysteine (Invitrogen), followed by incubation in the same medium supplemented with 50 μM L-azidohomoalanine (Invitrogen). The cells were then washed three times with warm PBS and lysed with lysis buffer (1% SDS; 50 mM Tris-HCl, pH 8.0) and a protease inhibitor cocktail (Sigma-Aldrich) for 30 minutes. Next, the lysates were sonicated to solubilize the proteins and disperse the DNA and subsequently centrifuged at 17,000 × g at 4°C for 5 minutes. The supernatants were then transferred to a clean tube, and the protein concentration was determined using a BCA protein assay kit (Pierce). Next, azide-labeled proteins were added to alkyne-containing biotin via chemoselective ligation between an azide and an alkyne by using a click-it protein reaction buffer kit (Invitrogen) according to the manufacturer's instructions.

Nuclear and cytoplasmic proteins extraction

At least 4×10^6 PMs or RAW264.7 cell lines were used for nuclear and cytoplasmic proteins extraction by NE-PER nuclear and cytoplasmic extraction reagents (Thermo) according to the manufacturer's instructions. GAPDH and histone H3 were used as cytoplasmic and nuclear loading controls respectively.

Histological analysis

Lungs and livers from control or VSV-infected mice were dissected, fixed in 4% paraformaldehyde, embedded into paraffin, cut into sections, stained with hematoxylin and eosin solution, and examined by light microscopy for histology changes. Immunohistochemical staining with anti-STAT1 antibody in wild-type and *NDR1*-deficient murine livers was performed using standard procedures.

Immunofluorescence staining and microscopy

RAW264.7 overexpressing flag-NDR1 cells cultured on cover slips were fixed for 10 min with 4% PFA and permeabilized for 10 min at room temperature with 0.1% Triton X-100. After incubation for blockade of nonspecific binding for 30 min, anti-flag antibody was added and incubated for overnight at 4°C. Samples were further stained with suitable Alexa Fluor 488–conjugated secondary antibodies (Thermo Fisher Scientific) for 1 h at room temperature. Images were acquired on an Olympus IX81-FV1000 fluorescence microscope (Olympus).

Supplementary Table 1. List of antibody information.

Antibody	Clone	Distributor	Dilution
anti-β-actin	AA128	beyotime	1:2000
anti-GAPDH	M1211-1	epitomics	1:2500
anti-NDR1	H00011329-M02	novusbio	1:1000
anti-STAT1	sc-346	Santa Cruz Biotechnology	1:1000
anti-Biotin	sc-101339	Santa Cruz Biotechnology	1:1000
Anti-phospho-STAT1	#9171	Cell Signaling Technology	1:1000
Anti-phospho-ERK	#4370	Cell Signaling Technology	1:1000
Anti-phospho-JNK	#4668	Cell Signaling Technology	1:1000
Anti-phospho-p38	#9216	Cell Signaling Technology	1:2500
Anti-phospho-TBK1	#5483	Cell Signaling Technology	1:1000
Anti-phospho-IRF3	#4947	Cell Signaling Technology	1:1000
Anti-phospho-p65	#3033	Cell Signaling Technology	1:1000
anti-ERK	#4696	Cell Signaling Technology	1:1000
anti-JNK	#9295	Cell Signaling Technology	1:1000
anti-P38	#8690	Cell Signaling Technology	1:3000
anti-TBK1	#3504	Cell Signaling Technology	1:1000
anti-P65	#8242	Cell Signaling Technology	1:1000
anti-STAT2	#72604	Cell Signaling Technology	1:500
anti-STAT3	#9139	Cell Signaling Technology	1:2000
anti-STAT5	#9363	Cell Signaling Technology	1:1000
anti-STAT6	#9397	Cell Signaling Technology	1:1000
anti-JAK1	#3344	Cell Signaling Technology	1:500
anti-Histone H3	#3638	Cell Signaling Technology	1:2500
anti-IRF3	11312-1-AP	proteintech	1:3000
anti-RIG-I	20566-1-AP	proteintech	1:2000
anti-STAT1	10144-2-AP	proteintech	1:1000
anti-IRAK1	10478-2-AP	proteintech	1:1000
anti-TRAF6	R1311-2	HUABIO	1:1000
anti-flag	0912-1	HUABIO	1:1000
anti-flag	#M20008	Abmart	1:2500
anti-myc	#M20002	Abmart	1:2500
anti-HA	#M20003	Abmart	1:2500
anti-LC3B	L7543	Sigma-Aldrich	1:1000
anti-H3K4me3	07-473	Millipore	1:100
anti- H3K27me3	07-449	Millipore	1:100

Supplementary Table 2. List of primers for ChIP-qPCR.

mouse miR146a intergenic primer 1	Forward	GGAGCATTGTCAACCCCAGT
	Reverse	CCTTCGGTCTGGCTGAATCT
mouse miR146a intergenic primer 2	Forward	AGCAGAGTCCAAAAGACAAAAGG
	Reverse	AGAAGCTGTCCGGTTCTCTG
mouse miR146a intergenic primer 3	Forward	GCCACCATCCGGACCA
	Reverse	CTCTGGCGCACCCTCTAAC
mouse miR146a regulatory sequences primer A	Forward	ACTTGGGGATACAAGCAGCG
	Reverse	GTGGTTAGGGGTCTTGCCTG
mouse miR146a regulatory sequences primer B	Forward	AGATGGATGCTTGGGAACGA
	Reverse	CAGGGGAACAAAGAGGTGCTT
mouse miR146a regulatory sequences primer C	Forward	CCCTCTCCAGAGGCCAATTC
	Reverse	CGTAGGCAGAGTTTCGACCA
mouse miR146a regulatory sequences primer D	Forward	GGATTTCCCCACAGGCTAGG
	Reverse	GACCCTCTCTTAACCAGGCG
mouse miR146a regulatory sequences primer E	Forward	TGCTGGCTTTAAGGTGGGAG
	Reverse	CTCAATTCCCTCCTCGTGGC
mouse miR146a regulatory sequences primer F	Forward	TCTTCTGGAAGGCTCTTGGC
	Reverse	GCCTGGCTGGTTTTACCACT
mouse IL6 promoter primer	Forward	GCAGTGGGATCAGCACTAAC
	Reverse	GGTGGGTAAAGTGGGTGAAG
mouse TNFα promoter primer	Forward	CAGCCACTGCTTGGCTAGAC
	Reverse	CGGATCCCATGGACCAACTG
mouse IL1β promoter primer	Forward	CTACCTTTGTTCCGCACATCC
	Reverse	CCCTCCCTTGTTTTATAGTCC
human NDR1 promoter primer	Forward	CCGCTGGGTAACCTCCCAAA
	Reverse	CCCCCTACCCCGTGTAGAA
human miR146a promoter primer	Forward	CATTGGGCAGCCGATAAAGC
	Reverse	TCTAGAGCGTTCTGTGCAGG

Supplementary Table 3. List of the detailed information of RSV-infected patients.

Number	Gender	Age	Grade of infection	respiratory tract virus antigens tests					other respiratory tract infection tests				clinical symptom		
				RSV	metapneumovirus	parainfluenza virus	influenza virus	adenovirus	Legionella pneumophila	Mycoplasma pneumonia	Chlamydia pneumoniae	tuberculosis	Cough	Short of breath	Fever
1	male	2M7D	moderate	+	−	−	−	−	−	−	−	−	+	+	−
2	male	1Y3M	mild	+	−	−	−	−	−	−	−	−	+	+	+
3	female	2M21D	mild	+	−	−	−	−	−	−	−	−	+	+	+
4	male	2M29D	moderate	+	−	−	−	−	−	−	−	−	+	+	−
5	male	1M4D	moderate	+	−	−	−	−	−	−	−	−	+	+	−
6	male	1M19D	mild	+	−	−	−	−	−	−	−	−	+	+	−
7	female	2Y4M	mild	+	−	−	−	−	−	−	−	−	+	−	+
8	male	5M15D	mild	+	−	−	−	−	−	−	−	−	+	+	−
9	male	4M4D	moderate	+	−	−	−	−	−	−	−	−	+	+	−
10	female	4M21D	moderate	+	−	−	−	−	−	−	−	−	+	+	−
11	male	2M29D	moderate	+	−	−	−	−	−	−	−	−	+	+	−
12	male	1M10D	moderate	+	−	−	−	−	−	−	−	−	+	+	−
13	male	1M4D	severe	+	−	−	−	−	−	−	−	−	+	+	−
14	female	2M26D	mild	+	−	−	−	−	−	−	−	−	+	+	−
15	male	2M16D	mild	+	−	−	−	−	−	−	−	−	+	+	−
16	male	1M20D	moderate	+	−	−	−	−	−	−	−	−	+	+	−
17	male	7M27D	mild	+	−	−	−	−	−	−	−	−	+	+	−
18	male	6M15D	mild	+	−	−	−	−	−	−	−	−	+	+	−
19	male	2M24D	severe	+	−	−	−	−	−	−	−	−	+	+	−
20	male	3M13D	mild	+	−	−	−	−	−	−	−	−	+	+	−
21	female	6M20D	mild	+	−	−	−	−	−	−	−	−	+	+	+
22	female	1M21D	severe	+	−	−	−	−	−	−	−	−	+	+	+
23	male	5M1D	severe	+	−	−	−	−	−	−	−	−	+	+	−
24	female	2M11D	mild	+	−	−	−	−	−	−	−	−	+	+	−
25	female	4M12D	mild	+	−	−	−	−	−	−	−	−	+	+	−
26	male	10M1D	mild	+	−	−	−	−	−	−	−	−	+	+	−
27	male	1M24D	mild	+	−	−	−	−	−	−	−	−	+	+	−
28	female	2M10D	mild	+	−	−	−	−	−	−	−	−	+	+	−
29	female	1M23D	moderate	+	−	−	−	−	−	−	−	−	+	+	−
30	male	1M14D	moderate	+	−	−	−	−	−	−	−	−	+	+	−
31	male	2M6D	severe	+	−	−	−	−	−	−	−	−	+	+	−
32	male	3M20D	moderate	+	−	−	−	−	−	−	−	−	+	+	−
33	male	2M24D	moderate	+	−	−	−	−	−	−	−	−	+	+	−
34	female	6M13D	mild	+	−	−	−	−	−	−	−	−	+	+	−
35	male	1M20D	moderate	+	−	−	−	−	−	−	−	−	+	+	−
36	female	2M26D	mild	+	−	−	−	−	−	−	−	−	+	+	−
37	female	2M2D	moderate	+	−	−	−	−	−	−	−	−	+	+	−

38	female	3M19D	mild	+	-	-	-	-	-	-	-	-	+	+	-
39	male	6M8D	mild	+	-	-	-	-	-	-	-	-	+	+	-
40	female	10M14D	moderate	+	-	-	-	-	-	-	-	-	+	+	-
41	female	1M6D	moderate	+	-	-	-	-	-	-	-	-	+	+	-
42	female	3M5D	mild	+	-	-	-	-	-	-	-	-	+	+	-
43	female	1M16D	moderate	+	-	-	-	-	-	-	-	-	+	+	-
44	male	4M14D	moderate	+	-	-	-	-	-	-	-	-	+	+	-
45	male	2M4D	moderate	+	-	-	-	-	-	-	-	-	+	+	-
46	male	1M11D	mild	+	-	-	-	-	-	-	-	-	+	+	-
47	male	4M18D	mild	+	-	-	-	-	-	-	-	-	+	+	+
48	male	3M3D	mild	+	-	-	-	-	-	-	-	-	+	+	-
49	male	2M3D	mild	+	-	-	-	-	-	-	-	-	+	+	-
50	male	5M16D	moderate	+	-	-	-	-	-	-	-	-	+	+	-
51	male	8M17D	mild	+	-	-	-	-	-	-	-	-	+	+	-
52	male	9M9D	mild	+	-	-	-	-	-	-	-	-	+	+	-
53	male	2M11D	mild	+	-	-	-	-	-	-	-	-	+	+	-
54	male	9M9D	mild	+	-	-	-	-	-	-	-	-	+	+	-
55	male	1M15D	moderate	+	-	-	-	-	-	-	-	-	+	+	-
56	male	2M8D	mild	+	-	-	-	-	-	-	-	-	+	+	-
57	female	1M9D	moderate	+	-	-	-	-	-	-	-	-	+	+	-
58	male	2M21D	mild	+	-	-	-	-	-	-	-	-	+	+	-
59	female	2M24D	moderate	+	-	-	-	-	-	-	-	-	+	+	+
60	male	1M4D	mild	+	-	-	-	-	-	-	-	-	+	+	-
61	female	1M8D	mild	+	-	-	-	-	-	-	-	-	+	+	-
62	male	4M2D	mild	+	-	-	-	-	-	-	-	-	+	+	-
63	female	1M28D	mild	+	-	-	-	-	-	-	-	-	+	+	-
64	male	5M18D	moderate	+	-	-	-	-	-	-	-	-	+	+	-
65	male	1M20D	moderate	+	-	-	-	-	-	-	-	-	+	+	-
66	male	2M20D	mild	+	-	-	-	-	-	-	-	-	+	+	-
67	male	2M2D	mild	+	-	-	-	-	-	-	-	-	+	+	-
68	male	2M5D	mild	+	-	-	-	-	-	-	-	-	+	+	-
69	male	4M19D	mild	+	-	-	-	-	-	-	-	-	+	+	-
70	male	11M12D	mild	+	-	-	-	-	-	-	-	-	+	+	+
71	female	9M23D	mild	+	-	-	-	-	-	-	-	-	+	+	-
72	female	2M5D	moderate	+	-	-	-	-	-	-	-	-	+	+	-
73	male	3M27D	mild	+	-	-	-	-	-	-	-	-	+	+	-
74	male	1M5D	mild	+	-	-	-	-	-	-	-	-	+	+	-
75	male	1M24D	moderate	+	-	-	-	-	-	-	-	-	+	+	-
76	male	1M9D	moderate	+	-	-	-	-	-	-	-	-	+	+	-
77	female	2M1D	moderate	+	-	-	-	-	-	-	-	-	+	+	-

In the age group, "Y" is short for year, "M" is for "month","D" is for "day"

Supplementary Table 4. List of gRNAs for CRISPR-CAS9.

human NDR1 1# guide for cas9	Forward	ACCGGCTCGTTGGCTGCCGCCGC
human NDR1 1# insert for cas9	Reverse	AACGCGGCGGCAGCCAACGAGCC
human NDR1 2# guide for cas9	Forward	ACCGCTAAACCTCTACCTAATCA
human NDR1 2# insert for cas9	Reverse	AACTGATTAGGTAGAGGTTTAGC
human miR146a 1# guide for cas9	Forward	ACCGTTTATAACTCATGAGTGCC
human miR146a 1# insert for cas9	Reverse	AACGGCACTCATGAGTTATAAAC
human miR146a 2# guide for cas9	Forward	ACCGCAGGGACTGCTGAGGCTTC
human miR146a 2# insert for cas9	Reverse	AACGAAGCCTCAGCAGTCCCTGC
mouse miR146a 1#guide for cas9	Forward	ACCGCACATGTATGAGGGCACAG
mouse miR146a 1# insert for cas9	Reverse	AACCTGTGCCCTCATACATGTGC
mouse miR146a 2# guide for cas9	Forward	ACCGGCACCGGAGGGAGTCATGT
mouse miR146a 2# insert for cas9	Reverse	AACACATGACTCCCTCCGGTGCC
mouse NDR1 DBS 1# guide for cas9	Forward	ACCGACCGCTGCTGCTGAGGCTG
mouse NDR1 DBS 1# insert for cas9	Reverse	AACCAGCCTCAGCAGCAGCGGTC
mouse NDR1 DBS 2# guide for cas9	Forward	ACCGCCCTGGCTGTCCCCCTTGA
mouse NDR1 DBS 2# insert for cas9	Reverse	AACTCAAGGGGGACAGCCAGGGC
human NDR1 cas9 identification primer	Forward	CCGGGAAGTCGCGCAT
	Reverse	AGTTTCACATGGCCCTTGCT
human miR146a cas9 identification primer	Forward	GTGTCTACCATACACATCCCCT
	Reverse	TGTTTCTCATCTTCTTTCACTGC
mouse miR146a cas9 identification primer	Forward	GGTCTTGCTGAGGAGGTGATTC
	Reverse	CTTGACGATGTATGAGACGAGCTG
mouse NDR1 DBS cas9 identification primer	Forward	CAGCCTGCAAAGGGTACTGC
	Reverse	CCCAAAGACTTGCAAAGCCA

Supplementary Table 5. List of primers for qPCR.

mouse β-actin	Forward	AACAGTCCGCCTAGAAGCAC
	Reverse	CGTTGACATCCGTAAAGACC
mouse TNFα	Forward	CTGGGACAGTGACCTGGACT
	Reverse	GCACCTCAGGGAAGAGTCTG
mouse IL6	Forward	AGTTGCCTTCTTGGGACTGA
	Reverse	TCCACGATTTCCCAGAGAAC
mouse IFNβ	Forward	ACACCAGCCTGGCTTCCATC
	Reverse	TTGGAGCTGGAGCTGCTTATAGTTG
mouse NDR1	Forward	AAGGGCCATGTGAAACTTTCC
	Reverse	CAGGAGTGCCCACTGTAGAGA
mouse STAT1	Forward	TCACAGTGGTTCGAGCTTCAG
	Reverse	GCAAACGAGACATCATAGGCA
mouse MX1	Forward	GACCATAGGGGTCTTGACCAA
	Reverse	AGACTTGCTCTTTCTGAAAAGCC

mouse MX2	Forward	GAGGCTCTTCAGAATGAGCAAA
	Reverse	CTCTGCGGTCAGTCTCTCT
mouse ISG15	Forward	GGTGTCCGTGACTAACTCCAT
	Reverse	TGGAAAGGGTAAGACCGTCCT
mouse IFIT1	Forward	AGTACAACGAGTAAGGAGTCACT
	Reverse	AGGCCAGTATGTTGCACATGG
mouse IFIT2	Forward	CTGAGATGTCACTTCACATGGAA
	Reverse	GTGCATCCCCAATGGGTTCT
mouse SOCS1	Forward	CCTGGTTGTAGCAGCTTGT
	Reverse	CGACCCCTGGTTTGTGCAA
mouse NOS2	Forward	CAGATCCCGAAACGCTTCA
	Reverse	TGTTGAGGTCTAAAGGCTCCG
mouse CXCL9	Forward	CTTTTCCTTTTGGGCATCATCT
	Reverse	GCAGGAGCATCGTGCATTC
mouse CXCL10	Forward	CCTGCCCACGTGTTGAGAT
	Reverse	TGATGGTCTTAGATTCCGGATTC
VSV	Forward	ACGGCGTACTTCCAGATGG
	Reverse	CTCGGTTCAAGATCCAGGT
HSV-1	Forward	TGGGACACATGCCTTCTTGG
	Reverse	ACCCTTAGTCAGACTCTGTTACTTACCC
HCV	Forward	CGGGAGAGCCATAGTGG
	Reverse	AGTACCACAAGGCCTTTCG
human GAPDH	Forward	GAGTCAACGGATTTGGTCGT
	Reverse	GACAAGCTTCCCGTTCTCAG
human TNFα	Forward	CCAGACCAAGGTCAACCTCC
	Reverse	CAGACTCGGCAAAGTCGAGA
human IL6	Forward	AGAGGCACTGGCAGAAAACAAC
	Reverse	AGGCAAGTCTCCTCATTGAATCC
human IFNβ	Forward	TTGTTGAGAACCTCCTGGCT
	Reverse	TGACTATGGTCCAGGCACAG
human NDR1	Forward	TCAGCACATGCTCGGAAGG
	Reverse	ACAAGCCGTACCTCACCAAAT
human MX1	Forward	GTTTCCGAAGTGGACATCGCA
	Reverse	CTGCACAGGTTGTTCTCAGC
human ISG15	Forward	CGCAGATCACCCAGAAGATCG
	Reverse	TTCGTCGCATTTGTCCACCA
human IFIT1	Forward	TTGATGACGATGAAATGCCTGA
	Reverse	CAGGTCACCAGACTCCTCAC
U6	Forward	CTCGCTTCGGCAGCACA
	Reverse	AACGCTTCACGAATTTGCGT
miR146a	Forward	TCGGTGAGAACTGAATTCCA
	Reverse	GTGCAGGGTCCGAGGT
human pri-miR146a	Forward	TGAGAACTGAATTCCATGGGTT

	Reverse	ATCTACTCTCTCCAGGTCCTCA
human pre-miR146a	Forward	CCGATGTGTATCCTCAGCTTTG
	Reverse	GCTGAAGAACTGAATTTCAGAGGTC
mouse pri-miR146a	Forward	TGAGAACTGAATTCCATGGGTTA
	Reverse	ACAGAGCTATCCCAGCTGAAG
mouse pre-miR146a	Forward	TCTGAGAACTGAATTCCATGGGTTA
	Reverse	AGCTGAAGAACTGAATTTCACAGGT

Supplementary references

1. Palleroni AV, Varesio L, Wright RB, Brunda MJ. Tumoricidal Alveolar Macrophage and Tumor Infiltrating Macrophage Cell-Lines. *Int J Cancer* **49**, 296-302 (1991).

2. Zhou HW, Imrich A, Kobzik L. Characterization of immortalized MARCO and SR-AI/II-deficient murine alveolar macrophage cell lines. *Part Fibre Toxicol* **5**, (2008).

3. Ma F*, et al.* Retinoid X receptor alpha attenuates host antiviral response by suppressing type I interferon. *Nature communications* **5**, 5494 (2014).

4. Bai R, Weng C, Dong H, Li S, Chen G, Xu Z. MicroRNA-409-3p suppresses colorectal cancer invasion and metastasis partly by targeting GAB1 expression. *Int J Cancer* **137**, 2310-2322 (2015).

5. Szenthe K*, et al.* The 5 ' regulatory sequences of active miR-146a promoters are hypomethylated and associated with euchromatic histone modification marks in B lymphoid cells. *Biochem Bioph Res Co* **433**, 489-495 (2013).

6. Sheng J, Yu W, Gao X, Xu Z, Hu GF. Angiogenin stimulates ribosomal RNA transcription by epigenetic activation of the ribosomal DNA promoter. *Journal of cellular physiology* **229**, 521-529 (2014).
